# Heteroleptic Oxidovanadium(V) Complexes with Activity against Infective and Non-Infective Stages of *Trypanosoma cruzi*

**DOI:** 10.3390/molecules26175375

**Published:** 2021-09-03

**Authors:** Gonzalo Scalese, Ignacio Machado, Gustavo Salinas, Leticia Pérez-Díaz, Dinorah Gambino

**Affiliations:** 1 Área Química Inorgánica, Facultad de Química, Universidad de la República, Montevideo 11800, Uruguay; gscalese@fq.edu.uy; 2 Programa de Posgrados de la Facultad de Química, Universidad de la República, Montevideo 11800, Uruguay; 3 Área Química Analítica, Facultad de Química, Universidad de la República, Montevideo 11800, Uruguay; imachado@fq.edu.uy; 4 Worm Biology Lab, Institut Pasteur de Montevideo, Montevideo 11400, Uruguay; gsalinas@pasteur.edu.uy; 5 Departamento de Biociencias, Facultad de Química, Montevideo 11800, Uruguay; 6 Laboratorio de Interacciones Moleculares, Facultad de Ciencias, Universidad de la República, Montevideo 11400, Uruguay; lperez@fcien.edu.uy

**Keywords:** vanadium, 8-hydroxyquinoline derivatives, *Trypanosoma cruzi*, metallomics, trypomastigotes

## Abstract

Five heteroleptic compounds, [V^V^O(IN-2H)(L-H)], where L are 8-hydroxyquinoline derivatives and IN is a Schiff base ligand, were synthesized and characterized in both the solid and solution state. The compounds were evaluated on epimastigotes and trypomastigotes of *Trypanosoma cruzi* as well as on VERO cells, as a mammalian cell model. Compounds showed activity against trypomastigotes with IC_50_ values of 0.29–3.02 μM. IN ligand and the new [V^V^O_2_(IN-H)] complex showed negligible activity. The most active compound [V^V^O(IN-2H)(L2-H)], with L2 = 5-chloro-7-iodo-8-hydroxyquinoline, showed good selectivity towards the parasite and was selected to carry out further biological studies. Stability studies suggested a partial decomposition in solution. [V^V^O(IN-2H)(L2-H)] affects the infection potential of cell-derived trypomastigotes. Low total vanadium uptake by parasites and preferential accumulation in the soluble proteins fraction were determined. A trypanocide effect was observed when incubating epimastigotes with 10 × IC_50_ values of [V^V^O(IN-2H)(L2-H)] and the generation of ROS after treatments was suggested. Fluorescence competition measurements with DNA:ethidium bromide adduct showed a moderate DNA interaction of the complexes. In vivo toxicity study on *C. elegans* model showed no toxicity up to a 100 μM concentration of [V^V^O(IN-2H)(L2-H)]. This compound could be considered a prospective anti-*T. cruzi* agent that deserves further research.

## 1. Introduction

Chagas’ disease or American trypanosomiasis is a neglected tropical disease (NTD-WHO), which is endemic in Latin America but has spread in recent decades to non-endemic regions owing to the migration of unaware infected people. Its etiological agent is the homoflagellated protozoan parasite *Trypanosoma cruzi* (*T. cruzi*)*,* whose metacyclic trypomastigote form is transmitted to mammalian host by blood-sucking triatomine bugs. Infective non-replicative metacyclic trypomastigotes, released in the feces during the insect blood meal, enter the mammalian host through skin wounds or mucosal membranes, invade mammalian cells and develop into the intracellular replicative amastigote form. Amastigotes differentiate back into highly motile trypomastigotes that are released upon cell lysis to blood circulation. Released trypomastigotes can then infect other host cells or be transmitted to the insects during their feeding. Inside the insect, they differentiate to rapidly dividing epimastigotes that remain in the insect gut and later convert into metacyclic trypomastigotes restarting the life cycle. Other modes of transmission are through blood transfusion, organ transplant and transmission from mother to fetus. Currently, there are around seven million infected people, 10,000 annual deaths and approximately 25 million people living in risk zones, mainly in rural regions of Latin America [1,2,3,4,5,6].

Available chemotherapy is based on Benznidazole and Nifurtimox, two drugs developed more than 50 years ago, which proved to be toxic, require a long treatment as compared to the rate of disease progression, and often develop drug resistance. Although many natural and synthetic compounds have been assayed for activity against *T. cruzi*, very few have successfully progressed through clinical trials [2,5,7,8,9].

In recent decades, Medicinal Inorganic Chemistry has proven to be a promising approach in the search for new therapeutic tools against Neglected Tropical Diseases (NTDs), particularly against Chagas disease [10,11,12,13,14,15,16,17,18,19,20,21].

Vanadium is a biologically relevant bioelement and many vanadium-based therapeutic drugs have been proposed for the treatment of several types of diseases, mainly diabetes and cancer. Oxidovanadium(IV) and (V) moieties, V^IV^O^2+^, V^V^O_2_^+^ and V^V^O^3+^, have been the most relevant in vanadium medicinal chemistry, although oligomeric forms have also shown interesting biological activity. Biochemical studies have demonstrated that vanadium compounds exert their action through mechanisms that mainly involve the generation of radical oxygen species (ROS) and inhibition of enzymes, especially certain regulatory phosphatases [21,22,23,24,25,26,27,28,29].

In the last 15 years our group has dedicated significant efforts to the rational design of vanadium-based compounds as antiproliferative agents against *T. cruzi* [17,21]. Different series of V^IV^O-based compounds bearing activity against *T. cruzi* were developed by including polypyridyl ligands that have DNA intercalating capacity [17,21]. In particular, heteroleptic compounds including dideprotonated tridentate salicylaldehyde semicarbazones as coligands showed promising results [30,31,32]. Later on, the research was expanded by choosing the bidentate 8-hydroxyquinoline scaffold for substituting the NN ligands. The quinoline moiety is an important heterocyclic pharmacophore that possesses a diversity of biological properties. 8-hydroxyquinoline (8HQ) is considered a “privileged structure” in Medicinal Chemistry since it is a versatile chemical scaffold that deserves to be further exploited for therapeutic applications. It can be modified and functionalized leading to derivatives, which may display favorable drug-like properties providing families of bioactive compounds [33,34,35]. In particular, 8HQ and some of its derivatives have shown antiparasitic activity and 8HQ metal complexes have demonstrated different biological activities [36,37,38,39,40]. Four bioactive oxidovanadium(V) heteroleptic compounds including 8HQ were developed and their biological behavior on *T. cruzi* was studied in detail [41]. More recently, two homoleptic series [V^IV^O(L-H)_2_] and [V^V^O(OCH_3_)(L-H)_2_], with L = 8-hydroxyquinoline derivatives, were studied in depth to shed light into the significance of the presence of the tridentate co-ligand on the biological behavior of the vanadium compounds [42]. Globally, results demonstrated the importance of the presence and nature of the tridentate coligand. Therefore, a new tridentate ligand IN was designed and obtained by condensation of the drug isoniazid and 2-hydroxy-1-naphtaldehyde.

**Figure 1:**
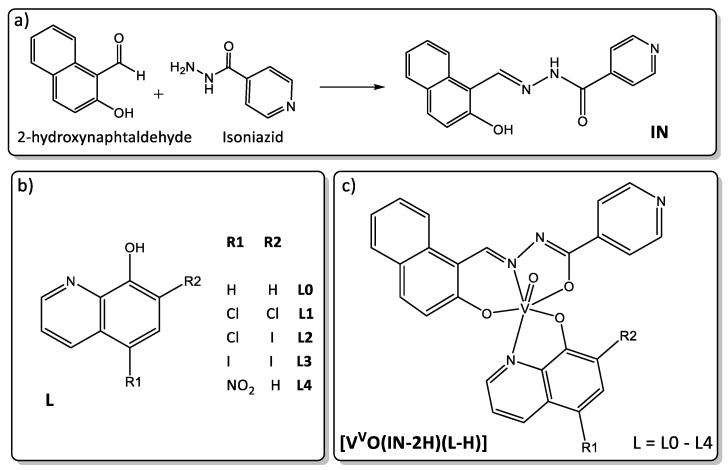
(**a**) Scheme of synthesis of the new IN tridentate ligand; (**b**) structural formulae of the selected 8-hydroxyquinoline derivatives L0–L4; (**c**) molecular structure of the new [V^V^O(IN-2H)(L-H)] complexes.

In the current work, a new series of five [V^V^O(IN-2H)(L-H)] compounds, where L are 8-hydroxyquinoline derivatives L0-L4 and IN is the new tridentate ligand, were synthesized and fully characterized (Figure 1). The biological activity of the whole series of oxidovanadium compounds and the ligands was evaluated against epimastigote and trypomastigote forms of *T. cruzi* as well as on VERO cells, as a mammalian cell model, for determining selectivity of the compounds towards the parasites.

In addition, the effect of the most active compound of the series on *T. cruzi*’s infection process and persistence was studied by evaluating the performance of pre-treated cell-derived trypomastigotes in the infection process and in the proliferation of treated intracellular amastigotes in a pre-established infection, respectively. To study the metallomics of this hit compound, the percentage of vanadium uptaken by the parasite and the preferred association of the compound with parasite biomacromolecules were also determined. Trying to obtain insight into the mechanism of action of the compounds, the generation of ROS in the parasite and DNA interaction were studied. Finally, the toxicity of the hit compound was evaluated using *Caenorhabditis elegans* (*C. elegans*), a nematode model organism that is used for drugs screening as a simplified proxy of animal toxicity.

## 2. Results and Discussion

A new tridentate ligand was synthesized by condensation of 2-hydroxynaphtaldehyde and isoniazid and characterized in the solid state and in solution by different techniques.

Five new heteroleptic oxidovanadium(V) complexes including the tridentate IN ligand and 8-hydroxyquinoline derivatives L0–L4 (Figure 1) were obtained with reasonable yields. V^V^O-complexes were obtained in THF starting from [V^IV^O(acac)_2_] and using equimolar ratio of reactants. Under the synthesis conditions, vanadium(IV) undergoes oxidation by molecular oxygen as previously reported for the homoleptic V^V^O-hydroxyquinoline derivatives [42,43].

Elemental analyses, FTIR and NMR spectroscopic and XRD results are in agreement with the proposed formula for IN free ligand and the five new [V^V^O(IN-2H)(L-H)] complexes. Their molecular formulae agree with those depicted in Figure 1. Moreover, analytical, FTIR and NMR spectroscopic results for the new IN dioxidovanadium(V) complex [V^V^O_2_(IN-H)], synthesized for comparative purposes, agree with the proposed formula [44,45,46].

### 2.1. Characterization in the Solid State

#### 2.1.1. FTIR Results

All [V^V^O(IN-2H)(L-H)] complexes showed a similar spectroscopic behavior. Based on our previous reports on vibrational behavior of heteroleptic oxidovanadium(V) complexes and homoleptic oxidovanadium(IV) and (V) complexes with 8-hydroxyquinoline derivatives and reports for other related complexes, selected IR bands of the new [V^V^O(IN-2H)(L-H)] complexes were identified and tentatively assigned (Supplementary Materials Table S1) [31,41,42,43,44,45,46,47,48,49].

IN free ligand presents a strong band at 1680 cm^−1^ corresponding to the ν(C=O) stretching that is absent in the complexes, indicating the enolization of the amide functionality upon coordination to the metal center. Instead, bands at ca. 1620 cm^−1^ and 1593 cm^−1^ are observed which are characteristic of a single CO bond typical of the coordination of the ligand enolate form (Figure S1) [31,32,41,46,50]. The simultaneous presence of the semicarbazone ligand L and the 8-hydroxyquinoline derivative co-ligand in the coordination sphere of vanadium led to quite complex spectra, particularly in the 1600–1500 cm^−1^ region (Figures S2–S6), where bands due to ν(C=C) and ν(C=N) of heterocyclic compounds usually appear. These bands are slightly shifted to lower wavenumbers when compared to the free ligands, suggesting V–N bond formation [42,43,45]. The shift of ν(C=O) and ν(C=N) bands and the non-observation of the ν(O-H) (in the 3026–3235 cm^−1^ region) and the ν(N-H) (3038 cm^−1^ in IN free ligand) bands upon complexation are in agreement with tridentate coordination of IN through the carbonylic oxygen (O_C=O_), the azomethyne nitrogen (N_azomethyne_) and the phenolic oxygen (O_phenolate_), and with deprotonation of the IN ligand at the phenolic hydroxyl and the NH group. Additionally, the results suggest that L ligand acts as monodeprotonated bidentate ligand. All [V^V^O(IN-2H)(L-H)] complexes showed a characteristic intense band around 966–978 cm^−1^ assigned to ν(V=O) (Table S1).

The FTIR pattern of the new [V^V^O_2_(IN-H)] compound was similar to that previously reported for similar dioxidovanadium(V) complexes with semicarbazone ligands (Figure S7) [41,46,49,51,52]. The shift of ν(C=O) and ν(C=N) bands and the non-observation of the ν(OH) are in agreement with tridentate coordination through the carbonylic oxygen, the azomethyne nitrogen and the phenolic oxygen, and with deprotonation of the semicarbazone ligand at the phenolic hydroxyl group. Additionally, the complex showed two bands assigned to the symmetric and antisymmetric stretchings of the VO_2_^+^ moiety.

#### 2.1.2. X-ray Diffraction Study of IN and [V^V^O(IN-2H)(L2-H)]·1.5 THF

**Figure 2:**
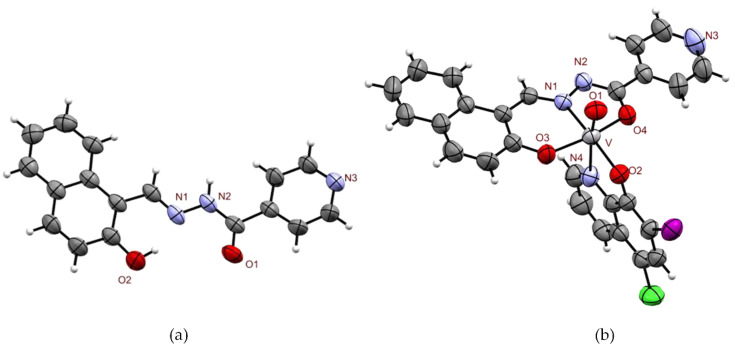
ORTEP drawing depicting a view of: (**a**) IN free ligand; (**b**) [V^V^O(IN-2H)(L2-H)]·1.5 THF showing the labeling of the O and N atoms and the displacement ellipsoids at the 50% probability level (solvent molecules were removed to clarify the visualization).

ORTEP drawings of IN free ligand and [V^V^O(IN-2H)(L2-H)]·1.5 THF complex are shown in Figure 2. Bond distances and angles around vanadium(V) in [V^V^O(IN-2H)(L2-H)]·1.5 THF are depicted in Table S2.

Data set of the IN free ligand suggests that the *E* form is stabilized. The crystal lattice is not stabilized by intermolecular H-bond, showing only intramolecular H-bond between the azomethyne N and the phenolic H (*d*(N1-H) = 1.739 Å).

The results show that the oxidovanadium(V) complex is an octahedral monomeric unit containing the doubly deprotonated IN ligand and monodeprotonated L2 in the coordination sphere. The tridentate IN ligand occupies three equatorial positions coordinating through carbonylic oxygen in its enolate form (*d*(V-O4) = 1.931(4) Å), deprotonated phenolic oxygen (*d*(V-O3) = 1.845(4) Å) and azomethine nitrogen (*d*(V-N1) = 2.077(4) Å). On the other hand, the L2 ligand is coordinated through deprotonated phenolic oxygen occupying the fourth equatorial position (*d*(V-O2) = 1.853(4) Å) and through nitrogen occupying one of the axial positions (*d*(V-N4) = 2.390(4) Å). The octahedron is completed with the oxo ligand occupying the second axial position (*d*(V-O1) = 1.590(4) Å).

Crystallographic data for the structures reported in this paper have been deposited in the Cambridge Crystallographic Data Centre (CCDC) as supplementary publication no. CCDC-1992304 for IN free ligand and CCDC-1992805 for [V^V^O(IN-2H)(L2-H)]·1.5 THF. Copies of the data can be obtained free of charge from the CCDC (12 Union Road, Cambridge CB2 1EZ, UK, http://www.ccdc.cam.ac.uk).

### 2.2. Characterization in Solution

#### NMR Results

The IN free ligand, the whole [V^V^O(IN-2H)(L-H)] series and the new [V^V^O_2_(IN-H)] complex were characterized in solution by ^1^H NMR and homonuclear and heteronuclear correlation experiments. The NMR spectra show narrow signals, typical of diamagnetic complexes (Figures S8–S14). Homonuclear and heteronuclear experiments allowed the assignment of all ^1^H resonances for the studied complexes. The assignment including chemical shifts (δ), multiplicities and coupling constants (*J*) for all studied compounds are presented after the description of the synthesis in the Section 3. Integrations of ^1^H NMR and signal multiplicities are in agreement with the proposed molecular formula. Tetrahydrofuran crystallization molecules in [V^V^O(IN-2H)(L2-H)] and [V^V^O(IN-2H)(L3-H)] were confirmed by NMR in agreement with the proposed formula. The ^1^H NMR chemical shift values along with the chemical shift differences between each complex and the corresponding ligand (expressed as Δδ) for the [V^V^O(IN-2H)(L-H)] complexes are listed in Table S3. The figure depicted in the table shows the general numbering scheme of IN and L. The ligands L0-L4 are shown in Figure 1b. The assignment of L0–L4 free ligands were extracted from the literature [53].

The five [V^V^O(IN-2H)(L-H)] complexes show similar ^1^H chemical shifts of the coordinated IN ligand and the L common protons in each molecule. In addition, they show a similar pattern of chemical shifts changes to the homoleptic [V^V^O_2_(IN-H)], shown in its synthesis description (Section 3.2.2), and to the previously reported analogous heteroleptic oxidovanadium(V) complexes with salicylaldehyde semicarbazone derivatives and 8-hydroxyquinoline [41]. As expected, protons 8 and 9 are not observed in the spectra of the five [V^V^O(IN-2H)(L-H)] complexes, in agreement with the double deprotonation of the IN ligand. A missing signal corresponding to proton 14 suggests the monodeprotonation of the L ligands.

As it has been discussed for previous oxidovanadium(V) complexes [41], upon coordination the de-shielding effect of the metal is apparent in some protons near the metal center (i.e., protons 1 and 20), causing a down-field shift of the corresponding ^1^H NMR peaks. The up-field shift may be the result of a decreasing azomethyne anisotropic effect in the coordinated form of IN. This effect has been also observed for the previously reported complexes with semicarbazone ligands [41,44,46].

### 2.3. Biological Results

#### 2.3.1. In Vitro Activity against Epimastigotes and Trypomastigotes of *T. cruzi* and Selectivity towards the Parasites

The five new [V^V^O(IN-2H)(L-H)] complexes were evaluated in vitro for their anti- *T. cruzi* activities against epimastigotes and cell-derived trypomastigotes of CL Brener strain. The results were compared to those of the reference drug Nifurtimox, the free ligands and the [V^V^O_2_(IN-H)] complex. Table 1 shows the whole set of results and Figure S15 shows a graphical comparision of IC_50_ values on both stages of *T. cruzi*.

The newly synthesized [V^V^O(IN-2H)(L-H)] complexes (L = L1–L4) showed IC_50_ values in the low micromolar range on epimastigotes (IC_50_ = 3.45–7.70 µM), with IC_50_ values of the same order as those of Nifurtimox and the free L. Trypomastigotes were more sensitive to the new heteroleptic complexes than epimastigotes, showing IC_50_ values seven to 70 times lower than the reference drug (IC_50_ = 0.29–3.02 µM). Selectivity towards the parasite for the series [V^V^O(IN-2H)(L-H)], the [V^V^O_2_(IN-H)] complex, and free L was tested by evaluating the unspecific cytotoxicity of all these compounds on VERO cells as mammalian cell model. The IC_50_ values on VERO cells together with the selectivity index values (SI = IC_50_ VERO cells/IC_50_ *T. cruzi*) are depicted in Table 1. The selectivity of the complexes towards the parasite increases significantly when substituents on the hydroxyquinoline moiety are included, as they are particularly favored by the inclusion of halogen substituents.

Although a clear trend to increase the selectivity index through the inclusion of the ligands in these heteroleptic compounds is not observed, the new compounds show interesting anti-*T. cruzi* activities in the low micromolar and submicromolar range, and some of them show a good selectivity towards the parasites. [V^V^O_2_(IN-H)] and IN free ligand showed negligible activity on both parasite stages, with IC_50_ values of the same order as their unspecific cytotoxicity, as they are non-selective compounds.

Since the [V^V^O(IN-2H)(L2-H)] complex was the most active compound of the series (IC_50_ trypomastigotes = 0.29 µM; SI = 109), it was selected to carry out further assays in order to explore its biological behavior.

#### 2.3.2. Stability Studies and Active Species

To shed light on the chemical changes that the studied compounds may suffer after disolution, NMR stability studies of the complexes in DMSO—the solvent used to prepare a stock solution for the biological trials—were performed.

**Figure 3:**
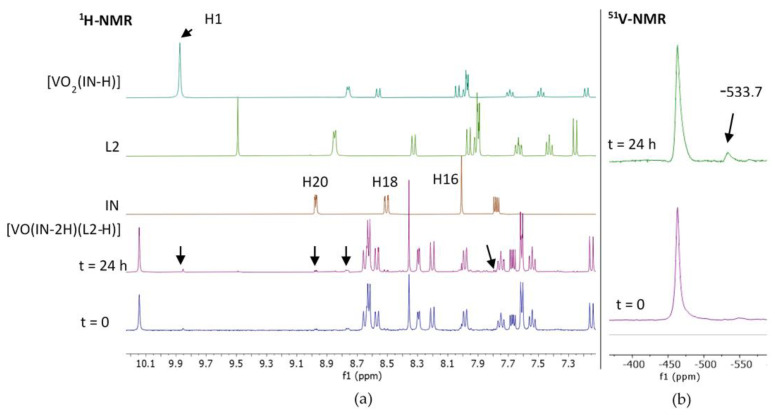
(**a**) ^1^H-NMR of [V^V^O(IN-2H)(L2-H)] in DMSO-*d*_6_ (23.5 mM) at t = 0 and 24 h, compared with ^1^H-NMR spectra of free ligands and [V^V^O_2_(IN-H)]. Selected signals were labeled and pointed with an arrow in the spectra of the heteroleptic complex. (**b**) ^51^V-NMR of [V^V^O(IN-2H)(L2-H)] in DMSO-*d*_6_ (23.5 mM) at t = 0 and 24 h.

Both ^1^H and ^51^V NMR spectra of the most active compound of the series [V^V^O(IN-2H)(L2-H)] were measured in DMSO-*d*_6_ at initial time (30–45 min after dissolution) as well as 24 h after that. At the initial time, ^1^H NMR spectra show minimal signals related to decomposition which slightly increase with time (Figure 3a). Particularly, after 24 h the spectra show signals of free L2, very small signals from free IN and signals from the homoleptic compound [V^V^O_2_(IN-H)]. Figure 3a follows the signal of H1 in the homolepctic compound at chemichal shift δH1= −9.85 ppm. Additionally, ^51^V NMR shows a unique species with δV = −463 ppm. A second signal appears after 24 h at δ = −533.7 ppm, integrating 7% respect to the original one (Figure 3b). According to previous reports of related systems, the species responsible of this new signal could be ascribed to [V^V^O_2_(IN-H)] [32,41,54]. This finding suggests a very small descomposition of the [V^V^O(IN-2H)(L2-H)], mainly leading to [V^V^O_2_(IN-H)] and free L2.

Thus, the observed biological activity is likely to be due predominantely to the effect of the original compound but partially to released L, since [V^V^O_2_(IN-H)] has negligible activity. The whole set of results shows that the mode of decomposition of this new complexes is in agreement with the results by Levina and Lay and by ourselves for heteroleptic oxidovanadium(IV) complexes including tridentate O,N,O-ligands and a bioactive co-ligand and for the series [V^V^O(L-2H)(8HQ-H)], where L are semicarbazones derivatives [32,41,55,56].

#### 2.3.3. Effects on the *T. cruzi* Infection Process and Persistance

**Figure 4:**
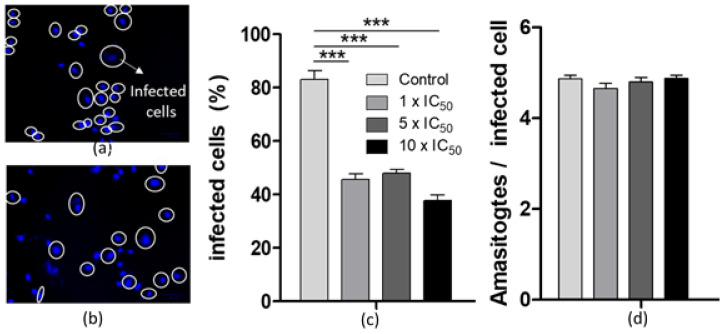
Effect of [V^V^O(IN-2H)(L2-H)] on the infection process after 24 h post-incubation. Infection established with (**a**) untreated cell-derived trypomastigotes (control); (**b**) 1 × IC_50_ treatment. White circles show infected cells (Infected cells were stained with DAPI and counted for each treatment). (**c**) Percentage of infected cells using pretreated cell-derived trypomastigotes. Comparison of infected cell percentages with control untreated cell-derived trypomastigotes (control) was performed using the one-way ANOVA test, followed by the Bonferroni post hoc test. (**d**) Number of amastigotes per infected cell after 24 h of incubation with the indicated concentrations of the complex. The mean and SD are shown for each condition. Three independent experiments were performed. In each experiment, at least 300 cells were counted. ANOVA test: *** = *P* < 0.001.

In order to investigate the effect of the most active compound of the series on the parasite’s infection process, cell-derived trypomastigotes were incubated for 30 min with [V^V^O(IN-2H)(L2-H)] in concentrations of 1 ×, 5 × and 10 × the IC_50_ value previously determined on cell-derived trypomastigotes (Table 1). Pre-treated trypomatigotes were then used to infect VERO cells. The number of infected cells was evaluated after 24 and 48 h for each treatment and compared with control cells infected with untreated trypomastigotes (Figure 4a–c). After both tested times, the percentage of infected cells decreases statistically significantly. The effect tends to be dose-dependent after 48 h post-infection with treated trypomastigotes.

In addition, to evaluate the effect of the vanadium compound on the proliferation of intracellular amastigotes, a pre-established infection was treated with 1 ×, 5 × and 10 × the IC_50_ value previously determined on cell-derived trypomastigotes. The number of amastigotes per infected cell was counted for each treatment. For all treatments, no decrease in the amount of amastigotes per cell was observed after both incubation times tested (Figure 4d). This finding suggests that the new [V^V^O(IN-2H)(L2-H)] complex affects the infection process, but it does not affect the ability of amastigotes to proliferate intracellularly at the concentrations studied.

#### 2.3.4. Parasite Recovery Results

Parasite recovery experiments were performed to investigate if the V^V^O-complex displays a trypanostatic or trypanocide effect on the epimastigote form. The effect of the compound was considered trypanostatic if the parasites resume growth after a period of 96 h in fresh medium, or trypanocide when it leads to an irreversible arrest of cell growth.

To be able to compare the obtained whole set of results, epimastigotes were incubated with concentrations of the compound corresponding with 1 ×, 5 × and 10 × the IC_50_ value previously determined on cell-derived trypomastigotes (0.29, 1.45 and 2.90 µM, respectively).

**Figure 5:**
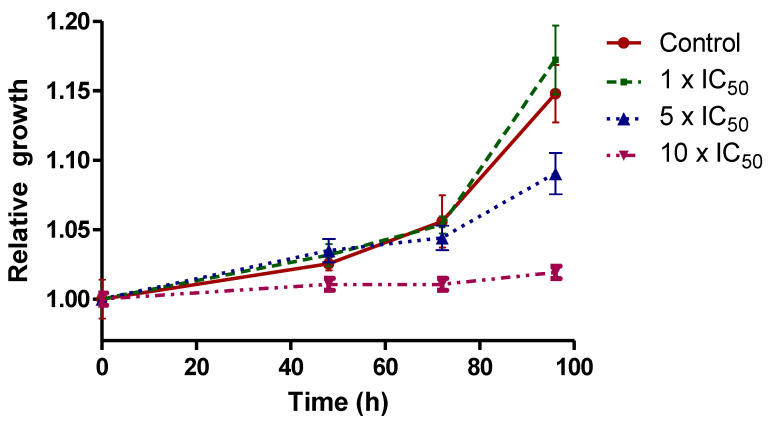
Recovery assays in fresh culture medium after incubation of the parasites with the [V^V^O(IN-2H)(L2-H)] complex for 24 h. Each experiment was performed in triplicate and the mean and standard deviation is represented for each point.

Similar parasite growth recovery profiles were observed in untreated parasites and parasites treated with the two lowest doses, suggesting a trypanostatic effect at these concentrations. On the other hand, for the 10 × IC_50_ treatment, a trypanocide effect could be suggested, since treated parasites could not recover the growth profile even after 96 h of recovery in fresh medium without compound (Figure 5).

As previously described for similar oxidovanadium(V) complexes, the compound may exert a dual type of activity depending on the concentration employed, displaying a trypanostatic effect at low concentrations and a trypanocide effect at higher ones [30,41,42].

#### 2.3.5. Vanadium Uptaken by Epimastigotes and Trypomastigotes

Vanadium uptaken by the parasites or strongly bound (not removable by washing) and vanadium remaining in the culture medium were determined by electrothermal atomic absorption spectrometry in order to estimate the amount of compound into both forms of the parasites. As far as we know, no uptake values have been previously reported for vanadium compounds or other metal compounds in trypomastigotes of *T. cruzi*.

Epimastigotes and trypomastigotes were incubated with [V^V^O(IN-2H)(L2-H)] for 4 and 24 h at concentrations of 1 ×, 5 × and 10 × the IC_50_ value previously determined on cell-derived trypomastigotes (0.29, 1.45 and 2.90 µM, respectively). The average amount of vanadium determined for three independent experiments is shown in Table 2.

As presented in Table 2, the metal incorporation percentages do not change significantly between 4 and 24 h in both epimastigotes and trypomastigotes. The uptake percentages are quite low when compared with those previously reported in epimastigotes for [V^V^O(8HQ-H)(L’-2H)], where L’ is the tridentate 2-hydroxy-1-naphthaldehyde semicarbazone derivative and for [V^IV^O(L1-H)_2_], where L1 is 5,7-dichloro-8-hydroxyquinolne [41,42]. On the other hand, they are of the same order as those of the previous hit compound [V^IV^O(L-2H)(NN)], with L = 5-bromosalicylaldehyde semicarbazone and NN = 5-aminophenanthroline [30]. This differential uptake could arise from the different chemical nature of the compounds and/or different speciation in incubation media [56]. Our results show that in trypomastigotes, the uptaken percentage is higher than in epimastigotes. This finding could partially explain the IC_50_ differences between both stages of the parasite (see Table 1).

#### 2.3.6. Vanadium Association with Parasite Macromolecules

**Figure 6:**
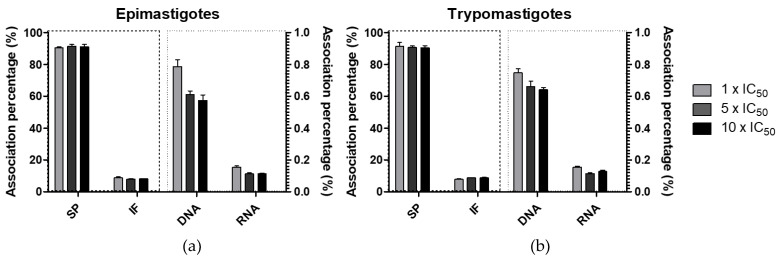
Percentage of vanadium associated with the different isolated macromolecules of (**a**) epimastigotes; (**b**) trypomastigotes, after 24 h of treatment with 1 ×, 5 × and 10 × the calculated IC_50_ value of [V^V^O(IN-2H)(L2-H)] on trypomastigotes of *T. cruzi*. The average and SD of independent triplicates of determinations in DNA, RNA, soluble (SP) and insoluble fraction (IF) are shown.

Vanadium associated with macromolecules was determined in order to analyze the subcellular distribution of the chosen compound, [V^V^O(IN-2H)(L2-H)], in the parasites. Epimastigotes and trypomastigotes were incubated for 24 h with the V^V^O-complex and then, different macromolecules (DNA, RNA and proteins) were isolated and total vanadium associated with each fraction was quantified by electrothermal atomic absorption spectrometry (Figure 6).

A similar pattern of vanadium distribution was observed in epimastigotes and trypomastigotes after 24 h of treatment with 1 ×, 5 × and 10 × the IC_50_ value previously determined on cell-derived trypomastigotes. Indeed, no statistically significative differences were found between incubation with different compound concentrations (ANOVA with Bonferroni’s post hoc test for multiple comparisons).

Less than 1% was found in nucleic acids fractions for all treatments on both stages. A preferential association to the soluble proteins fraction was observed with an average of 90.5%. Similar preferential association with soluble proteins was previously shown by [V^V^O(8HQ-H)(L’-2H)], where L’ is 2-hydroxy-1-naphthaldehyde semicarbazone, [V^IV^O(L1-H)_2_] and [V^IV^O(L’-2H)(NN)], where L’ is 5-bromosalicilaldehyde and NN is 5-amino-phenanthroline [30,41,42]. The fact that the association pattern is quite different in the previous systems indicates that in spite of the fact that the complexes probably undergo changes/speciation in the incubation media and several fractions of the parasite, the nature of the ligands does have significant effects, showing that the species in both cases are different.

#### 2.3.7. Generation of Reactive Oxygen Species

Trypanosomatids are particularly sensitive to reactive oxygen species (ROS) and possess specific defense systems against oxidative stress. Depending on the ROS levels produced, different cell death mechanisms might be induced [57,58]. ROS generation induced by vanadium compounds as a cytotoxic mechanism is well documented [24]. The higher ROS levels have been detected for complexes containing vanadium(IV) as metal center, but ROS generation has been also demonstrated for vanadium(V) compounds [59].

To elucidate whether [V^V^O(IN-2H)(L2-H)] complex induces ROS generation in the parasite, parasites were incubated with 1 ×, 5 × and 10 × the IC_50_ of the compound for 4 and 24 h. 2′,7′-dichlorodihydrofluorescein diacetate (H_2_DCFDA) was used to detect ROS. The cell-permeant H_2_DCFDA is a non-fluorescent chemically reduced form of fluorescein, that upon cleavage of the acetate groups by intracellular esterases and oxidation is converted to the highly fluorescent 2′,7′-dichlorofluorescein (DCF). Incubation with hydrogen peroxide (H_2_O_2_) leads to a loss of mitochondrial membrane potential and probably subsequent extensive lipid peroxidation in the parasite inducing necrosis [58,60]. Therefore, it was used as positive control.

**Figure 7:**
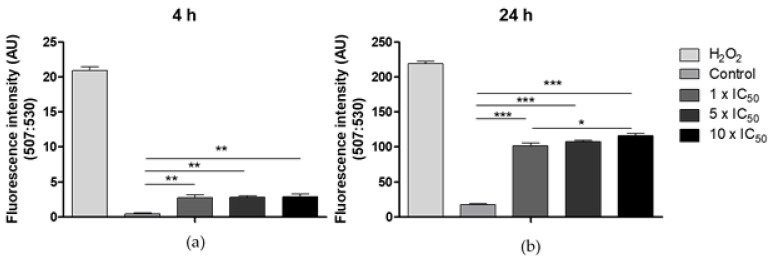
Analysis of ROS generation in epimastigotes, after (**a**) 4 h and (**b**) 24 h treatment with 1 ×, 5 × and 10 × the calculated IC_50_ value of [V^V^O(IN-2H)(L2-H)] on trypomastigotes of *T. cruzi*. The mean and SD are shown for each condition. Three independent experiments were performed. ANOVA test: * = *P* < 0.05, ** = *P* < 0.01, *** = *P* < 0.001.

All compound concentrations tested, increased significantly ROS levels compared to the control group (Figure 7). After 24 h a statistically significative increase in ROS production was also observed between the 1 × and 10 × IC_50_ concentrations.

#### 2.3.8. DNA Interaction by Fluorescence Studies

The interaction of the compound with DNA as a potential molecular target was followed employing ethidium bromide (EB), a planar fluorescent probe with a weak intrinsic fluorescence emission at the selected excitation wavelength of 510 nm. Under the selected experimental conditions, EB shows an emission maximum at 602 nm. The formation of the {DNA–EB} adduct by intercalation of EB into double stranded DNA induces an increase in the fluorescence quantum yield [29,61].

**Figure 8:**
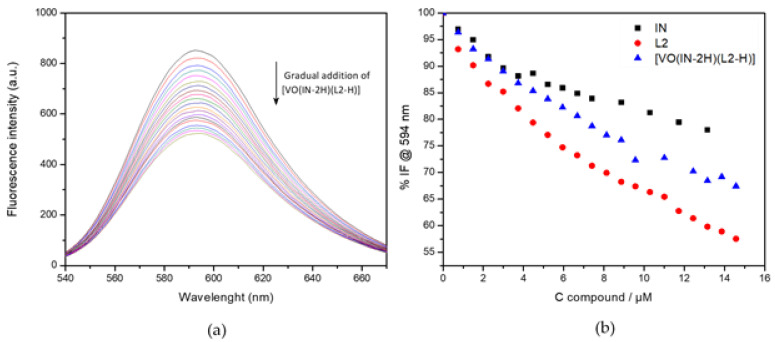
(**a**) Fluorescence quenching spectra (λexc = 510 nm) for {DNA–EB} adduct with increasing amounts of [V^V^O(IN-2H)(L2-H)]. (**b**) Relative fluorescence intensity (%) at λ = 594 nm with increasing compound concentration (C_DNA_ = 1.5 mM, C_EB_ = 0.75 mM, samples prepared in DMSO/buffer Tris HCl medium).

Under our experimental conditions, the emission maximum of the {DNA–EB} adduct is located at 594 nm. At the selected excitation wavelength, no fluorescence emission occurs from the direct interaction of [V^V^O(IN-2H)(L2-H)] or L2 and IN free ligands with DNA. A negligible emission at 594 nm was found due to direct interaction of the compounds with DNA, which was subtracted from the sample spectra. Complex and free ligands affect the emission of the {DNA–EB} adduct. A regular quenching in the emission of {DNA–EB} is observed upon increasing the concentration of the studied compounds, the extent of the quenching being of a similar order for IN, L2 and [V^V^O(IN-2H)(L2-H)] complex (Table 3). Changes in emission spectra of {DNA–EB} adduct while [V^V^O(IN-2H)(L2-H)] is gradually added are shown in Figure 8a as an example. Comparative results of the titration with the tested compounds are shown in Figure 8b.

The obtained *K*_SV_ values are of a similar order to those previously reported by us for the [V^IV^O(L-H)_2_] and [V^V^O(OCH_3_)(L-H)_2_] complexes, and other complexes showing antitrypanosomal activity [42,62,63,64]. Since *K*_SV_ is a direct measure of the efficiency of the whole quenching process and, indirectly, of the affinity of the tested molecules for ctDNA, we can conclude that the compounds show a quite low affinity for DNA. In addition, metallomics studies showed a negligible association of the complex to DNA fraction. All together, results allow DNA to be discarded as a main target of the compound.

#### 2.3.9. In Vivo Toxicity on *C. elegans*

**Figure 9:**
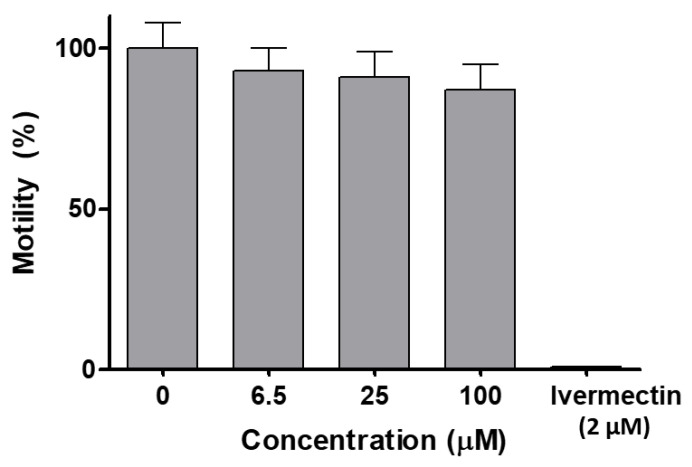
Seventy L4 adult worms were incubated in M9 buffer, 1% DMSO and the vanadium compound at different concentrations (6.25–100 µM range). Vehicle control (without compound) was used as a reference of normal motility in 1% DMSO. Ivermectin was used as a positive control at 2 µM. In each experiment, four replicas were performed for each concentration. One experiment of three biological replicates is shown. The experiment was performed twice. Error bars correspond to standard deviations.

The dose–response study on *C. elegans* showed that [V^V^O(IN-2H)(L2-H)] did not affect worm motility (Figure 9). At a concentration as high as 100 µM, which is the maximal solubility of the compound at 1% DMSO, there was a marginal decrease in motility after 18 h, which was not statistically significant (unpaired t test). In contrast, the known anthelmintic drug ivermectin, used as a positive control, reduced motility to zero at 2 µM (Figure 9). The worms treated with compound [V^V^O(IN-2H)(L2-H)] at 100 µM or with vehicle only were recovered from wells and seeded on NGM plates and food was added to assess viability and development. Worms treated at 100 µM were all alive and showed normal development (L4–adult worm–egg–L1).

*C. elegans* (*Caenorhabditis elegans*), a nematode model organism, has been used as a simplified proxy of animal toxicity, and also used as model for drug discovery as well as in ecotoxicological studies [65]. [V^V^O(IN-2H)(L2-H)] did not significantly affect motility after 18 h treatment in the concentration range used (6.5–100 µM). In addition, neither viability nor development were affected after treatment. These results indicate that this compound is innocuous for this animal invertebrate model that allows acute toxicity and developmental and reproductive toxicity (DART) to be examined.

## 3. Materials and Methods

### 3.1. Materials

All common laboratory chemicals were purchased from commercial sources and used without further purification. Derivatives of 8-hydroxyquinoline, isoniazid and 2-hydroxynaphtaldehyde were purchased from Sigma Aldrich.

### 3.2. Synthesis of the New IN Ligand, the Oxidovanadium(V) Complexes [V^V^O(IN-2H)(L-H)] and the Dioxidovanadium(V) Complex [V^V^O_2_(IN-H)]

#### 3.2.1. Synthesis of the IN Ligand

The new IN ligand was prepared from an equimolar mixture of 2-hydroxynaphtaldehyde and isoniazid (Figure 1a). Isoniazid (411 mg, 3.0 mmol) was dissolved in 5 mL of methanol (MeOH) and a solution of 2-hydroxynaphtaldehyde (567.5 mg, 3.3 mmol) in 5 mL of MeOH was added dropwise. The mixture was heated at reflux for 4 h. The yellow solid obtained was isolated by centrifugation and recrystallized from boiling ethanol.

***IN***. Yield: 650 mg, 74%. Anal (%) calc. for C_17_H_13_N_3_O_2_: C, 70.09; H, 4.50; N, 14.42. Found: C, 70.36; H, 4.58; N: 14.49. FTIR (KBr/cm^−1^): 3219 *ν*(O-H), 3039 *ν*(N-H), 1679 *ν*(C=O), 1595 *ν*(C=N). ^1^H NMR [DMSO-*d*_6_, δ/ppm]: 12.53 (s, 1H), 12.41 (s, 1H), 9.49 (s, 1H), 8.84 (d, *J* = 5.44 Hz, 2H), 8.32 (d, *J* = 8.5 Hz, 1H), 7.96 (d, *J* = 9.0 Hz, 1H), 7.94–7.84 (m, 3H), 7.63 (ddd, *J* = 8.4, 6.8, 1.4 Hz, 1H), 7.43 (ddd, *J* = 8.0, 6.9, 1.0 Hz, 1H), 7.26 (d, *J* = 9.0 Hz, 1H).

#### 3.2.2. Syntheses of the Dioxidovanadium(V) Complex, [V^V^O_2_(IN-H)]·THF

The new [V^V^O_2_(IN-H)] complex was synthesized dissolving 0.19 mmol of IN (55 mg) in 3 mL of warm tetrahydrofuran (THF) and adding dropwise a solution of 0.19 mmol of [V^IV^O(acac)_2_] in 3 mL of THF. The mixture was refluxed for 2 h and then kept stirring at room temperature for 3 days. The brown solid formed was filtered off, washed three times with THF and dried under vacuum.

***[V^V^O_2_(IN–H)]·THF*** Yield: 36 mg, 43%. Anal (%) calc. for C_21_H_16_N_3_O_5_V: C, 56.64; H, 4.53; N, 9.44. Found: C, 56.97; H, 4.23; N: 9.60. FTIR (KBr/cm^−1^): 3074 *ν*(N-H), 1633 *ν*(C=O), 1597 *ν*(C=N), 915 *ν*(VO_2_)*asym*, 827 *ν*(VO_2_)*sym*. ^1^H NMR [DMSO-*d*_6_, δ/ppm]: 9.87(s, 1H), 8.76 (d, *J* = 5.44 Hz, 2H), 8.56 (d, *J* = 8.5 Hz, 1H), 8.17 (d, *J* = 8.9 Hz, 1H), 7.99–7.95 (m, 3H), 7.68 (ddd, *J* = 8.4, 6.9, 1.4 Hz, 1H), 7.50 (ddd, *J* = 7.9, 6.9, 0.94 Hz, 1H), 7.18 (d, *J* = 9.0 Hz, 1H).

#### 3.2.3. Synthesis of the Oxidovanadium(V) Complexes [V^V^O(IN-2H)(L-H)], L = L0–L4

The new [V^V^O(IN–2H)(L–H)] complexes, where L = L0–L4, were synthesized by the following procedure: 0.19 mmol of L (28 mg L0, 40 mg L1, 57 mg L2, 74 mg L3 or 36 mg L4), 0.19 mmol of IN (55 mg) and 0.19 mmol of [V^IV^O(acac)_2_] (50 mg) were suspended in 5 mL of hot THF. Immediately the solution turned reddish brown. The mixture was heated at reflux for 4 h. The dark solution obtained was cooled and stirred at room temperature for several days. The dark black-violet solid formed was filtered off, washed three times with 0.5 mL portions of THF and dried under vacuum for 24 h.

***[V^V^O(IN–2H)(L0–H)]*** Yield: 42 mg, 44%. Anal. calc. for C_27_H_20_N_4_O_4_V: C, 62.92; H, 3.91; N, 10.87; Found: C, 62.84; H, 3.90; N, 10.98. FTIR (KBr/cm^−1^): 1620 *ν*(C=O), 1594 *ν*(C=N), 974 *ν*(V=O). ^1^H NMR [DMSO-*d*_6_, δ/ppm]: 10.12 (s, 1H), 8.68 (d, *J* = 8.6 Hz, 1H), 8.62 (dd, *J* = 4.5, 1.6 Hz, 2H), 8.55 (d, *J* = 8.9, 1H), 8.22 (d, *J* = 9.0 Hz, 1H), 8.17 (d, *J* = 8.6 Hz, 1H), 7.98 (dd, *J* = 8.0, 1.3 Hz, 1H), 7.70–7.85 (m, 5H), 7.63–7.59 (m, 4H), 7.20 (s, 1H), 7,10 (d, *J* = 9.0 Hz, 1H).

***[V^V^O(IN–2H)(L1–H)]*** Yield: 27 mg, 25%. Anal. calc. for C_27_H_18_Cl_2_N_4_O_4_V: C, 55.50; H, 3.11; N, 9.59; Found: C, 55.44; H, 3.02; N, 9.33. FTIR (KBr/cm^−1^): 1618 *ν*(C=O), 1593 *ν*(C=N), 978 *ν*(V=O). ^1^H NMR [DMSO-*d*_6_, δ/ppm]: 10.12 (s, 1H), 9.09(d, *J* = 9.0, 1.7 Hz, 1H), 8.72 (d, *J* = 8.7 Hz, 1H), 8.61 (d, *J* = 8.5 Hz, 1H), 8.58–8.54 (m, 3H), 8.29 (s, 1H), 8.17 (d, *J* = 9.0 Hz, 1H), 7.94 (dd, *J* = 8.1, 1.3 Hz, 1H), 7.76–7.65 (m, 2H), 7.57–7.53 (m, 2H), 7.49 (d, *J* = 7.4 Hz, 1H), 7.40 (d, *J* = 8.8 Hz, 1H), 7.08 (d, *J* = 9.0 Hz, 1H).

***[V^V^O(IN–2H)(L2–H)]·1.5 THF*** Yield: 45 mg, 31%. Anal. calc. for C_33_H_30_ClIN_4_O_5.5_V: C, 50.56; H, 3.86; N, 7.15; Found: C, 50.32; H, 3.80; N, 7.20. FTIR (KBr/cm^−1^): 1618 *ν*(C=O), 1593 *ν*(C=N), 972 *ν*(V=O). ^1^H NMR [DMSO-*d*_6_, δ/ppm]: 10.14 (s, 1H), 8.66–8.60 (m, 3H), 8.57 (dd, *J* = 8.5, 1.3 Hz, 1H), 8.35 (s, 1H), 8.29 (d, *J* = 4.6 Hz, 1H), 8.20 (d, *J* = 9.0 Hz, 1H), 7.98 (d, *J* = 8.1 Hz, 1H), 7.74 (t, *J* = 7.7 Hz, 1H), 7.67 (dd, *J* = 8.5, 4.6 Hz, 1H), 7.63–7.59 (m, 2H), 7.54 (t, *J* = 7.5 Hz, 1H), 7.14 (d, *J* = 9.0 Hz, 1H).

***[V^V^O(IN–2H)(L3–H)]·THF*** Yield: 139 mg, 88%. Anal. calc. for C_31_H_26_I_2_N_4_O_5_V: C, 44.36; H, 3.12; N, 6.68; Found: C, 44.25; H, 3.27; N, 6.63. FTIR (KBr/cm^−1^): 1620*ν*(C=O), 1593 *ν*(C=N), 966 *ν*(V=O). ^1^H NMR [DMSO-*d*_6_, δ/ppm]: 10.14 (s, 1H), 8.70 (s, 1H), 8.68–8.60 (m, 2H), 8.40–8.32 (m, 1H), 8.25–8.16 (m, 2H), 7.98 (dd, *J* = 8.3, 1.3 Hz, 1H), 7.75 (ddd, *J* = 8.5, 7.0, 1.4 Hz, 1H), 7.67–7.59 (m, 3H), 7.59–7.47 (m, 1H), 7.14 (d, *J* = 9.0 Hz, 1H).

***[V^V^O(IN–2H)(L4–H)]*** Yield: 51 mg, 48%. Anal. calc. for C_27_H_19_N_5_O_6_V: C, 57.87; H, 3.42; N, 12.50; Found: C, 57.84; H, 3.42; N, 12.43. FTIR (KBr/cm^−1^): 1618*ν*(C=O), 1593 *ν*(C=N), 968 *ν*(V=O). ^1^H NMR [DMSO-*d*_6_, δ/ppm]: 10.18 (s, 1H), 9.15 (d, *J* = 9.1, 1.8 Hz, 1H), 8.78 (d, *J* = 8.7 Hz, 1H), 8.67 (d, *J* = 8.5 Hz, 1H), 8.62 (dd, *J* = 4.5, 1.6 Hz, 2H), 8.35 (s, 1H), 8.23 (d, *J* = 9.0 Hz, 1H), 8.00 (dd, *J* = 8.1, 1.3 Hz, 1H), 7.82–7.71 (m, 2H), 7.63–7.59 (m, 2H), 7.55 (d, *J* = 7.4 Hz, 1H), 7.46 (d, *J* = 8.8 Hz, 1H), 7.14 (d, *J* = 9.0 Hz, 1H).

### 3.3. Physicochemical Characterization

C, N and H analyses were carried out with a Thermo Scientific Flash 2000 elemental analyzer (Thermo, Delft, The Netherlands). The FTIR spectra (4000–400 cm^−1^) were measured as KBr pellets with a Shimadzu IRPrestige-21 instrument (Shimadzu, Kyoto, Japan). The 1D ^1^H and ^51^V NMR spectra were recorded in DMSO-*d*_6_ at 25 °C on a Bruker Avance III 400 MHz spectrometer (Bruker Biospin AG, Faellanden, Switzerland). The 2D homonuclear (^1^H,^1^H-COSY), and heteronuclear one-bond (^1^H,^13^C-HSQC) and multiple-bond (^1^H,^13^C-HMBC) correlation experiments were carried out with the same instrument. Chemical shifts are reported in ppm using the solvent residual peak as an internal standard for ^1^H NMR (δ_H_ 2.50 ppm) and ^13^C NMR (δ_C_ 39.52 ppm).

To obtain an initial insight into the stability of the complexes in DMSO, solvent used to prepare compounds stock solutions on the biological studies, the ^1^H and ^51^V-NMR spectra of the most active compound of the series, [VO(IN-2H)(L2-H)], were measured at time zero and after 24 h of dissolution.

### 3.4. X-ray Diffraction of IN and [V^V^O(IN–2H)(L2–H)]·1.5 THF

Single yellow crystals of IN free ligand and single violet crystals of [V^V^O(IN–2H)(L2–H)]·1.5 THF, suitable for X ray diffraction studies, were obtained by slow evaporation of an ethanol solution and a THF solution, respectively.

The measurements were performed on a Bruker D8 Venture diffractometer with multilayer mirror monochromated CuKα (λ = 1.54178 Å) radiation. X-ray diffraction intensities were collected and integrated with APEX2 v2014.5-0 (Bruker AXS Inc., Madison, WI, USA). These intensities were scaled and the data were corrected empirically for absorption (employing the multi-scan method) with SADABS V2016/2 (Bruker AXS) program [66]. The structures were solved by direct methods with the SHELXT 2014/5 [67] and the molecular model developed by alternated cycles of Fourier methods and full-matrix least-squares refinement SHELXL-2018/3 running under SHELXle [68]. A summary of crystal data, data collection procedure, structure determination methods and refinement results is listed in Table S4.

### 3.5. Biological Studies

#### 3.5.1. In Vitro Activity against *Trypanosoma cruzi* and Cytotoxicity on a Mammalian Cell Model (VERO Cells)

##### Parasite and Culture

All parasite experiments were carried out using *T. cruzi* CL Brener strain.

*Epimastigotes*. Epimastigote forms were obtained from axenic cultures, maintained in a brain–heart infusion medium (Oxoid, Basingstoke, Hampshire, UK) supplemented with 10% heat inactivated fetal bovine serum (FBS, Capricorn), penicillin (100 units per mL) and streptomycin (100 mg mL^−1^) at 28 °C and harvested during the exponential phase of growth.

*VERO cells.* VERO cells (ATCC CCL81, Manassas, VA, USA) were used as mammalian cell model for testing unspecific cytotoxicity. This cell lineage was isolated from kidney epithelial cells from African green monkeys and is widely used for phenotypic screening of drugs as it is regarded as a normal cell derived from epithelial tissue [69].

Cells were cultured in RPMI medium (Gibco, Grand Island, NY, USA) supplemented with 10% heat inactivated fetal bovine serum, penicillin (100 units/mL) and streptomycin (100 µg/mL) at 37 °C in a humidified 5% CO_2_ incubator. For maintenance, confluent cells were washed with PBS, incubated for 3 min with trypsin-EDTA (Gibco), diluted and re-plated.

*Infections.* Epimastigotes were grown until a high proportion of metacyclic trypomastigote parasites were observed (~10 days). Trypomastigote-rich cultures were incubated overnight with a monolayer of VERO cells in a 10:1 parasite:cell ratio in RPMI medium at 37 °C in a humidified 5% CO_2_ incubator. Extracellular parasites were removed the following day by aspirating cell culture media, washing the VERO cell monolayer three times with PBS, followed by addition of fresh complete RPMI. Trypomastigotes emerged from VERO cells (4–5 days) were used to set up new VERO cell infections [70].

*Trypomastigotes.* Cell-derived trypomastigotes were obtained from the supernant of infected VERO cells collected 72 h post established infection with cell-emerged trypomastigotes.

##### Compounds’ Treatment

The compounds were initially dissolved in DMSO (stock concentration). Freshly solutions were diluted in the culture medium to obtain the different concentrations tested. Throughout the experimental procedures, the concentration of DMSO never exceeded 1%, which is non-toxic for the protozoa [71].

##### In Vitro Activity against Epimastigotes and Trypomastigotes of *Trypanosoma cruzi*

To determine the IC_50_ value (50% growth inhibitory concentration), a new technique was developed adapting the one previously reported for *Mycobacterium tuberculosis* [72]. At first, 1 × 10^6^ parasites per well were seeded in black 96-well plates in BHI medium (epimastigotes) or RPMI (trypomastigotes) with increasing concentration of compounds for 24 h. Viability was tested using alamar Blue^TM^ (Thermo Fisher, Waltham, MA, USA), where resazurin is reduced to resofurin, a compound that is red in color and highly fluorescent. To each well, 10 μL of alamar Blue was added. Black plates were incubated for 3 h at 28 °C. Fluorescence (excitation 530 nm/emission 590 nm) was measured in a Thermo Scientific Varioskan^®^ Flash Multimode instrument (Waltham, MA, USA). Nifurtimox (Nfx) was used as the reference anti-*T. cruzi* drug. Dose–response curves were recorded and the IC_50_ values were determined using GraphPad Prism version 6.00 for Windows (GraphPad Software, San Diego, CA, USA). The results are presented as averages ± SD (standard deviation) of three independent biological replicates.

##### Cytotoxicity on VERO Cells

For the cytotoxicity assay, 10,000 cells per well were seeded in a 96-well plate in RPMI medium and were incubated at 37 °C in a 5% CO_2_ atmosphere. Once adhered to the plate, cells were incubated with the indicated compound concentrations for 24 h. Cell viability was assessed using MTT (3-(4, 5-dimethylthiazolyl-2)-2, 5-diphenyltetrazolium bromide) assay, where MTT is reduced by metabolically active cells to generate reducing equivalents such as NADH and NADPH, resulting in the formation of an intracellular purple formazan which can be solubilized by the addition of DMSO. Briefly, 20 μL of MTT 5 mg/mL were added to each well. Plates were incubated for 4 h at 37 °C in a 5% CO_2_ atmosphere. After incubation, the medium was removed and the cells were disrupted with 100 μL of DMSO. Plates were kept for 15 min with agitation and absorbance was measured at 570 nm in a Thermo Scientific Varioskan^®^ Flash Multimode instrument (Waltham, MA, USA). Each assay was performed three times [69].

#### 3.5.2. In Vitro Infection Assays

##### Effect on the Infection Process

Per well, 50,000 cells were seeded in a 24-well plate in RPMI medium and were incubated at 37 °C in a 5% CO_2_ atmosphere until they adhered to the plate. Cell-derived trypomastigotes were obtained from the supernatant of infected VERO cells collected 96 h post infection and treated with the most active compound of the series, [V^V^O(IN-2H)(L2-H)] for 30 min at concentrations corresponding to 1 ×, 5 × and 10 × the previously calculated IC_50_ on trypomastigotes of *T. cruzi*. Pre-treated parasites were used to infect the seeded VERO cells in a 10:1 parasite:cell ratio. Infected cells were maintained in the same conditions for 24 and 48 h, washed three times with 1× PBS to remove the remaining trypomastigotes, fixed with 4% paraformaldehyde for 30 min and stained with DAPI. ANOVA test with Bonferroni correction was performed by analyzing twelve well plates and eight independent fields for each experiment [42].

##### Effect on the Intracellular Parasite Replication

Per well, 50,000 infected cells were seeded in a 24-well plate. Once adhered to the plate, infections were treated with the most active compound of the series, [V^V^O(IN-2H)(L2-H)], for 24 and 48 h at concentrations corresponding to 1 ×, 5 × and 10 × the previously calculated IC_50_ on trypomastigotes of *T. cruzi*. After that, the cells were washed three times with 1× PBS to remove the remaining trypomastigotes, fixed with 4% paraformaldehyde for 30 min and stained with DAPI. The number of amastigotes per cell was determined to evaluate the intracellular parasite replication.

For both infection assays, three independent assays were performed and at least 40 cells were counted in eight randomly selected fields to discriminate between infected and non-infected cells (~300 cells per condition) to evaluate the % of infected cells after each treatment [42].

#### 3.5.3. Parasite Recovery Assays

To evaluate if the compounds cause a trypanocide or a trypanostatic effect, recovery assays were carried out for the most active compound of the series, [V^V^O(IN-2H)(L2-H)]. The assays were performed as previously described [73]. Epimastigotes were incubated for 24 h with concentrations corresponding to 1 ×, 5 × and 1 0× the previously calculated IC_50_ on trypomastigotes of *T. cruzi*. After this incubation period, vanadium compound was removed by washing parasites with fresh BHI and transferring them to fresh BHI free of compound. Proliferation of pre-treated parasites was evaluated through absorbance measurements at 595 nm in a Thermo Scientific Varioskan^®^ Flash Multimode instrument (Waltham, MA, USA) at 24 h, 48 h and five days. Untreated control parasites were used as control to calculate relative proliferation. Assays were performed three times independently. The effect of the compound was considered trypanocide if the parasites were unable to resume growth after 48 h of culture post-compound incubation. Otherwise, the effect was considered trypanostatic.

#### 3.5.4. Uptake of Vanadium by Parasites

The determinations of vanadium compound uptake percentage were performed as previously described [74]. Briefly, epimastigotes and trypomastigotes in a density of 1 × 10^7^ parasites mL^−1^ were incubated with concentrations corresponding to 1 ×, 5 × and 10 × IC_50_ on trypomastigotes of *T. cruzi* of [V^V^O(IN-2H)(L2-H)]. Parasites were collected at 4 h and 24 h after incubation with the most active compound. Each sample (1 × 10^7^ parasites) was centrifuged at 1000 *g* for 10 min. The supernatant containing uncaptured compound was separated from the pellet of parasites. The parasites in the pellet fraction were washed with 1 × Phosphate-buffered saline (PBS) and resuspended in 500 μL PBS. Both fractions, i.e., pellet and supernatant, were analyzed separately by electrothermal atomic absorption spectrometry in a spectrophotometer Thermo iCE 3500 (Thermo Fisher Scientific, Cambridge, UK). The uptake percentage of vanadium in the parasites was determined according to the following equation: % entry = P/P + S, where P corresponds to total ng of vanadium in the parasites (pellet), S corresponds to ng of vanadium in the supernatant and P + S is total ng of the metal incorporated in the experiment (supernatant plus pellet). Vanadium was quantified by electrothermal atomic absorption spectrometry with a spectrophotometer Thermo iCE 3500 (Thermo Fisher Scientific) equipped with a graphite furnace atomizer. The optimal temperatures for pyrolysis and atomization were 1300 and 2750 °C, respectively. A 1000 mg L^−1^ V standard solution (stock) was prepared from VOSO_4_·5H_2_O dissolved in 0.1% *v*/*v* nitric acid (HNO_3_). Calibration solutions were prepared by dilution of the stock solution using 0.1% *v*/*v* HNO_3_. Temperature program was optimized for a complete vaporization of the samples. The limit of detection (3 s criteria) was 0.15 µg L^−1^. Precision, expressed as RSD (%), was better than 5%. Trueness, expressed as V recoveries, was in the range 95–102%. Three independent experiments were performed for each concentration at each analyzed time point.

#### 3.5.5. Vanadium Association with Parasite Macromolecules

Vanadium association with different macromolecules (DNA, RNA, soluble proteins and insoluble fraction) was analyzed for [V^V^O(IN-2H)(L2-H)] by electrothermal atomic absorption spectrometry in a spectrophotometer Thermo iCE 3500 (Thermo Fisher Scientific) [41,42,74]. Insoluble fraction mainly contains insoluble proteins and membrane lipids among other insoluble molecules.

Mid exponential phase parasites were incubated with 1 ×, 5 × and 10 × the IC_50_ determined value on trypomastigotes of *T. cruzi*. After 24 h of incubation, macromolecules were isolated for further analysis. For DNA isolation, 3 × 10^7^ parasites were collected using Monarch Genomic DNA Purification Kit. For protein isolation, 3 × 10^7^ parasites were resuspended in 1 mL of Parasite Lysis Buffer containing Tris-Cl 10 mM pH 7.5, EDTA 1 mM, CHAPS 1%, glycerol 10%, Triton 0.5%, and Complete™ Protease Inhibitor Cocktail (Roche, Manheim, Germany). After 30 min of stirring on ice, the lysate was centrifugated at 20,000× *g* for 1 h at 4 °C. The soluble proteins were isolated from the supernatant and the insoluble ones from the pellet, which were resuspended in 0.5 mL of PBS for further analysis. Total RNA was isolated using Trizol reagent (Life Technologies, Gaithersburg, MD, USA) starting from 2 × 10^7^ parasites. Three independent experiments were performed for all vanadium analytical determinations [41,42,74].

Taking in account the volume of each macromolecule fraction, total ng of vanadium was calculated in each case. Associated percentages were calculated as follows: ng of vanadium in fraction x/total ng of vanadium, where x is a given macromolecule fraction and total ng of vanadium is the sum of vanadium ng in RNA fraction + vanadium ng in DNA fraction + vanadium ng in soluble protein fraction + vanadium ng in insoluble protein fraction.

### 3.6. Production of Reactive Oxygen Species (ROS)

In order to determine whether the studied compounds cause oxidative stress in the parasites, the production of ROS induced by the most active complex [V^V^O(IN-2H)(L2-H)] was evaluated adapting a previously reported procedure [75]. Epimastigotes (1 × 10^7^ parasites/mL) were incubated for 4 h and 24 h with concentrations corresponding to 1×, 5 × and 10 × the previously calculated IC_50._ After this period of incubation, parasites were incubated in 10 μg/mL H2DCFDA (Thermo Fisher) in PBS for 1 h at 28 °C. H_2_O_2_ (50 μM) was used as a positive control for the production of ROS. The levels of ROS were analyzed in a Thermo Scientific Varioskan^®^ Flash Multimode instrument (Waltham, MA, USA) using an excitation wavelength of 507 nm and an emission wavelength of 530 nm. Three independent experiments were performed. ANOVA test with Bonferroni correction was performed by analyzing twelve well plates and eight independent fields for each experiment

### 3.7. DNA Interaction by Fluorescence Studies

Experiments for competitive binding to calf thymus DNA (ctDNA, SIGMA, Type I, No. D-1501, St. Louis, MO, USA) between ethidium bromide (EB, SIGMA) and the compounds were carried out at room temperature in 10 mM Tris-HCl buffer at pH 7.4. Millipore water was used for the preparation of solutions of the complexes for the studies and for buffer solution. Fluorescence measurements were performed as titration using a fixed concentration of ctDNA and EB while gradually increasing the concentration of the [V^V^O(IN-2H)(L2-H)] complex, L2 and IN free ligands. ctDNA stock solutions were prepared by hydrating ctDNA in Tris-HCl buffer (1 mg mL^−1^, ~2 mM nuc^−1^). This solution was allowed to stand at 4 °C. The concentration of the stock solution was determined by UV spectrophotometry using the molar absorption coefficient ε (260 nm) = 6600 M^−1^cm^−1^nuc^−1^ [76]. An EB 0.5 mM solution was prepared in Tris-HCl buffer. Due to the low solubility of the complexes in aqueous media, DMSO was used to prepare concentrated stock solutions, followed by appropriate dilution. The content of DMSO did not exceed 5% *v*/*v* in the final samples. Samples were prepared with a total concentration of DNA and EB of 1.5 μM and 0.75 μM, respectively, varying the ratio of compound until the ratio of {DNA:EB} adduct—complex = 1:10. Fluorescence spectra were recorded from 540 nm to 680 nm at an excitation wavelength of 510 nm on a Shimadzu RF 5301 spectrofluorimeter (Kyoto, Japan) and in a 10.0 × 10.0 mm^2^ quartz cuvette. Samples with only compounds and ctDNA (without EB) were used as blanks and subtracted from the sample spectra.

To determine the competitive binding ability of the complexes, the Stern–Volmer quenching constant were determined using Equation (1) [77]:

*F*_0_/*F* = 1 + *K_SV_*[Q] = 1 + *k_q_*τ[Q] (1)

where *F*_0_ and *F* are the fluorescence intensities of {DNA:EB} adduct in the absence and presence of the compounds, respectively. *K_SV_* is the Stern–Volmer quenching constant, *k_q_* is the bimolecular quenching rate constant, [Q] is the concentration of the quencher, and t is the average lifetime of the biomolecule in the excited state (typically ca. 10^−8^ s for biomacromolecules) [78,79]. *K_SV_* were determined from the plot of *F*_0_/*F* versus [Q].

### 3.8. In Vivo Toxicity on C. elegans

The wild-type *Caenorhabditis elegans* (*C. elegans*) Bristol strain N2 and *Escherichia coli* (*E. coli*) OP50 strain were obtained from the Caenorhabditis Genomics Center (Minneapolis, MN, USA). Worms were maintained under standard conditions at 20 °C on Nematode Growth Media (NGM) agar plates seeded with *E. coli* OP50 as a source of food as previously described [80]. The method used to assess toxicity is based on worm motility according to previously described [81,82]. Briefly, the locomotor activity recording system, WMicrotracker^TM^ ONE (PhylumTech, Santa Fe, Argentina) detects infrared microbeam interruptions. Synchronized L4 *C. elegans* worms were removed from culture plates and washed three times with M9 buffer (3 g KH_2_PO_4_; 6 g Na_2_HPO_4_; 5 g NaCl; 1 mL 1 M MgSO_4_ per liter) by centrifugation at 1000 *g*. Worms in M9 1% DMSO were plated in 96-well flat microtiter plates (Deltalab, Barcelona, Spain). Approximately 70 worms per well were seeded in 60 µL M9 buffer containing 1% DMSO and their basal movement was measured for 30 min to normalize the movement activity for each well at the beginning of the assay. Then, compound [V^V^O(IN-2H)(L2-H)] dissolved in M9 buffer 1% DMSO was added to a final concentration range of 6.5–100 µM in a final volume of 100 µL per well. Vehicle alone (1% DMSO) and the anthelmintic drug ivermectin at 2 µM were used as used controls. The motility using WMicrotracker^TM^ ONE was measured for 18 h. Motility of worms with vehicle only after 18 h was considered for reference as 100% motility. Four replicas were performed for each concentration in 96-well plates. Three biological replicas (three plates) were performed.

## 4. Conclusions

A new series of five structurally related mixed-ligand complexes, [V^V^O(IN-2H)(L-H)], where L are 8-hydroxyquinoline derivatives and IN is a tridentate Schiff base ligand derived from isoniazid, were synthesized and characterized in the solid state and in solution. These new V^V^O-compounds showed activity against the epimastigote and trypomastigote forms of *T. cruzi* (CL Brener strain). Infective trypomastigotes were more sensitive to the new compounds than epimastigotes, showing IC_50_ values seven to seventy times lower than the reference drug Nifurtimox. The anti-trypanosomal activity depicted by the compounds showed a low dependence on the nature of the substituents on the hydroxyquinoline moiety. Although the in vitro selectivity towards the parasite was relatively low, the study performed on *C. elegans* (*Caenorhabditis elegans*), a nematode model organism that is been used as a simplified proxy of animal toxicity in drug screening, indicated that the hit compound [V^V^O(IN-2H)(L2-H)], with L2 = 5-chloro-7-iodo-8-hydroxyquinoline, is innocuous for this animal invertebrate model up to 100 µM dose. Based on these preliminary toxicological results a low in vivo toxicity of the hit compound could be expected. Stability studies performed by NMR showed a partial decomposition of [V^V^O(IN-2H)(L2-H)] with time in DMSO solution, leading to the inactive species [V^V^O_2_(L2-H)] and IN and to the bioactive 8HQ derivative. Metallomics studies on the trypomastigote form of the parasite were performed for the first time in this work. A low uptake of vanadium and a preferential accumulation in the soluble proteins fraction was observed. In an attempt to obtain insight into potential targets, generation of ROS and interaction with DNA were studied. The hit compound generates oxidative stress in a dose dependent manner. Although the compound shows in vitro interaction with DNA, metallomics study showed only a negligible localization of the compound in the DNA fraction, which allows to discard this biomolecule as an important target. A set of biological tests was performed to obtain a deeper insight into the biological processes triggered by the hit compound in *T. cruzi*. Depending on the dose the compounds act as trypanocide or trypanostatic. [V^V^O(IN-2H)(L2-H)] affects the infection process, but it does not affect the ability of intracellular amastigotes to proliferate at the concentrations studied.

The whole set of results is encouraging for performing further studies on the hit compound and for further developing of prospective antitrypanosomal compounds based on vanadium.

## Figures and Tables

**Table 1:** In vitro activity on *T. cruzi* (epimastigotes and trypomastigotes of CL Brener strain), cytotoxicity on mammalian cells (VERO cells) and selectivity towards the parasite (SI value).

Compound	IC_50_ VERO ± SD (μM)	IC_50_ Epimastigotes *T. cruzi* ± SD (μM)	SI ^a^	IC_50_ Trypomastigotes *T. cruzi* ± SD (μM)	SI ^b^
Nifurtimox	998.5 ± 90.6 ^c^	3.68 ± 0.71	271	20.10 ± 2.86	49.6
L0	10.1 ± 3.9 ^c^	9.42 ± 2.23	1.1	0.47 ± 0.07	21.5
L1	66.5 ± 13.1 ^c^	7.17 ± 1.07	9.3	2.25 ± 0.95	29.5
L2	64.7 ± 9.1 ^c^	2.76 ± 0.52	23.4	0.40 ± 0.08	162
L3	75.5 ± 16.3 ^c^	2.17 ± 0.35	34.8	1.10 ± 0.88	68.6
L4	12.5 ± 4.5 ^c^	2.58 ± 0.56	4.8	1.01 ± 0.73	12.3
IN	22.64 ± 3.7	>20	≤1	>20	≤1
[V^V^O(IN–2H)(L0–H)]	10.99 ± 2.1	>15	≤1	1.92 ± 0.33	5.7
[V^V^O(IN–2H)(L1–H)]	22.03 ± 7.4	3.45 ± 0.48	6.4	1.46 ± 0.05	15.1
[V^V^O(IN–2H)(L2–H)]	31.54 ± 3.4	3.99 ± 1.30	7.9	0.29 ± 0.08	109
[V^V^O(IN–2H)(L3–H)]	54.18 ± 4.0	7.70 ± 0.67	7.0	1.69 ± 0.07	32.1
[V^V^O(IN–2H)(L4–H)]	42.09 ± 9.4	5.55 ± 1.41	7.6	3.02 ± 0.98	14.0
[V^V^O_2_(IN-H)]	>50	>20	nd	>20	nd

SI ^a^: IC_50_ VERO/IC_50_ *T. cruzi* epimastigotes; SI ^b^: IC_50_ VERO/IC_50_ *T. cruzi* tripomastigotes; ^c^ data from reference [42]; nd: not determined.

**Table 2:** Calculated percentages of vanadium uptaken by the parasites for [V^V^O(IN-2H)(L2-H)].

Compound Concencentration (μM) ^b^	% Entry ^a^ ± SD			
	Epimastigotes		Trypomastigotes	
	4 h	24 h	4 h	24 h
1 × IC_50_	4.5 ± 0.4	4.4 ± 0.7	7.1 ± 0.4	8.6 ± 0.4
5 × IC_50_	2.4 ± 0.2	3.4 ± 0.1	3.0 ± 0.1	4.3 ± 0.1
10 × IC_50_	2.0 ± 0.4	3.6 ± 0.05	3.1 ± 0.1	5.0 ± 0.1

^a^ % entry: % of vanadium uptaken by the parasite (pellet) relative to total vanadium added to the parasite culture. ^b^ IC_50_ values on trypomastigotes of *T. cruzi* determined as described under Materials and Methods.

**Table 3:** Stern–Volmer constants for [V^V^O(IN-2H)(L2-H)], L2 and IN for the competitive binding to {DNA-EB} adduct.

Compound	log (*K*_SV_)
IN	4.29
L2	4.68
[V^V^O(IN-2H)(L2-H)]	4.51

## Data Availability

Data is contained within the article or supplementary material.

## Supplementary Materials

The following are available online; Table S1: Tentative assignment of selected IR bands of the [V^V^O(IN–2H)(L-H)] complexes. Bands for the free ligands L0–L4, IN and the [V^V^O_2_(IN–H)] are included for comparison. Band positions are given in cm^−1^, Table S2: Selected bond lengths [Å] and angles around vanadium(V) [°] in [V^V^O(IN-2H)(L2-H)]·1.5 THF, Table S3: ^1^H-NMR signals of the [V^V^O(IN–2H)(L–H)] complexes in DMSO-*d*_6_ at 25 °C (δ, ppm). Free ligands are included for comparison, Table S4: Crystal data and structure refinement results for IN and [V^V^O(IN–2H)(L2–H)]·1.5 THF, Figure S1: Infrared spectra of IN free ligand (4000–400 cm^−1^), Figure S2: Infrared spectra of [VO(IN-2H)(L0-H)] (4000–400 cm^−1^), Figure S3: Infrared spectra of [VO(IN-2H)(L1-H)] (4000–400 cm^−1^), Figure S4: Infrared spectra of [VO(IN-2H)(L2-H)] (4000–400 cm^−1^), Figure S5: Infrared spectra of [VO(IN-2H)(L3-H)] (4000–400 cm^−1^), Figure S6: Infrared spectra of [VO(IN-2H)(L4-H)] (4000–400 cm^−1^), Figure S7: Infrared spectra of [VO(IN-2H)(L4-H)] (4000–400 cm^−1^), Figure S8: 1H-NMR spectra for IN free ligand, Figure S9: 1H-NMR spectra) for [VO_2_(IN-H)], Figure S10: 1H-NMR spectra for [VO(IN-2H)(L0-H)], Figure S11: 1H-NMR spectra for [VO(IN-2H)(L1-H)], Figure S12: 1H-NMR spectra for [VO(IN-2H)(L2-H), Figure S13: 1H-NMR spectra for [VO(IN-2H)(L3-H)], Figure S14: 1H-NMR spectra for [VO(IN2-H)(L4-H)], Figure S15: IC_50_ values on epimastigotes and trypomastigotes of *T. cruzi* of the new V^V^O-complexes comparing to free ligands and reference drug Nifurtimox (Nfx).

## Sample Availability

Samples of the compounds are not available from the authors.
