# Supplementary material for: Heteroleptic Oxidovanadium(V) Complexes with Activity against Infective and Non-Infective Stages of Trypanosoma cruzi
Source: Molecules. 2021 Sep 3;26(17):5375. doi: 10.3390/molecules26175375 (PMC8433833; doi:10.3390/molecules26175375)

checkCIF/PLATON report

Structure factors have been supplied for datablock(s) cu_191120_gs_isonaft_0m_a

THIS REPORT IS FOR GUIDANCE ONLY. IF USED AS PART OF A REVIEW PROCEDURE FOR PUBLICATION, IT SHOULD NOT REPLACE THE EXPERTISE OF AN EXPERIENCED CRYSTALLOGRAPHIC REFEREE.

No syntax errors found.   CIF dictionary   Interpreting this report

Datablock: cu_191120_gs_isonaft_0m_a

```
Bond precision:  C-C = 0.0021 A          Wavelength=1.54178

Cell:            a=8.786(2)     b=10.252(2)          c=15.793(5)
                 alpha=90       beta=99.423(10)      gamma=90
Temperature:     273 K

                Calculated                 Reported
Volume          1403.3(6)                  1403.5(7)
Space group     P 21/c                     P 21/c
Hall group      -P 2ybc                    -P 2ybc
Moiety formula  C17 H13 N3 O2              ?
Sum formula     C17 H13 N3 O2              C17 H13 N3 O2
Mr              291.30                     291.30
Dx,g cm-3       1.379                      1.379
Z               4                          4
Mu (mm-1)       0.759                      0.759
F000            608.0                      608.0
F000'           609.87
h,k,lmax        11,13,20                   11,12,19
Nref            3058                       3009
Tmin,Tmax       0.809,0.889                0.639,0.754
Tmin'           0.781

Correction method= # Reported T Limits: Tmin=0.639 Tmax=0.754
AbsCorr = MULTI-SCAN

Data completeness= 0.984          Theta(max)= 80.185

R(reflections)= 0.0470( 2457)     wR2(reflections)= 0.1404( 3009)

S = 1.033                   Npar= 208
```

The following ALERTS were generated. Each ALERT has the format
test-name_ALERT_alert-type_alert-level.
Click on the hyperlinks for more details of the test.

Alert level C

```
PLAT241_ALERT_2_C High   'MainMol' Ueq as Compared to Neighbors of        C16 Check
PLAT242_ALERT_2_C Low    'MainMol' Ueq as Compared to Neighbors of        C13 Check
PLAT911_ALERT_3_C Missing FCF Refl Between Thmin & STh/L=    0.600          2 Report
```

Alert level G

```
PLAT199_ALERT_1_G Reported _cell_measurement_temperature ..... (K)        273 Check
PLAT200_ALERT_1_G Reported   _diffrn_ambient_temperature ..... (K)        273 Check
PLAT883_ALERT_1_G No Info/Value for _atom_sites_solution_primary .     Please Do !
PLAT912_ALERT_4_G Missing # of FCF Reflections Above STh/L=  0.600         36 Note
PLAT978_ALERT_2_G Number C-C Bonds with Positive Residual Density.          2 Info
PLAT992_ALERT_5_G Repd & Actual _reflns_number_gt Values Differ by          4 Check
```

```
   0 ALERT level A = Most likely a serious problem - resolve or explain
   0 ALERT level B = A potentially serious problem, consider carefully
   3 ALERT level C = Check. Ensure it is not caused by an omission or oversight
   6 ALERT level G = General information/check it is not something unexpected

   3 ALERT type 1 CIF construction/syntax error, inconsistent or missing data
   3 ALERT type 2 Indicator that the structure model may be wrong or deficient
   1 ALERT type 3 Indicator that the structure quality may be low
   1 ALERT type 4 Improvement, methodology, query or suggestion
   1 ALERT type 5 Informative message, check
```

Validation response form

Please find below a validation response form (VRF) that can be filled in and pasted into your CIF.

```
# start Validation Reply Form
_vrf_PLAT241_cu_191120_gs_isonaft_0m_a
;
PROBLEM: High   'MainMol' Ueq as Compared to Neighbors of        C16 Check
RESPONSE: ...
;
_vrf_PLAT242_cu_191120_gs_isonaft_0m_a
;
PROBLEM: Low    'MainMol' Ueq as Compared to Neighbors of        C13 Check
RESPONSE: ...
;
_vrf_PLAT911_cu_191120_gs_isonaft_0m_a
;
PROBLEM: Missing FCF Refl Between Thmin & STh/L=    0.600          2 Report
RESPONSE: ...
;
# end Validation Reply Form
```

It is advisable to attempt to resolve as many as possible of the alerts in all categories. Often the minor alerts point to easily fixed oversights, errors and omissions in your CIF or refinement strategy, so attention to these fine details can be worthwhile. In order to resolve some of the more serious problems it may be necessary to carry out additional measurements or structure refinements. However, the purpose of your study may justify the reported deviations and the more serious of these should normally be commented upon in the discussion or experimental section of a paper or in the "special_details" fields of the CIF. checkCIF was carefully designed to identify outliers and unusual parameters, but every test has its limitations and alerts that are not important in a particular case may appear. Conversely, the absence of alerts does not guarantee there are no aspects of the results needing attention. It is up to the individual to critically assess their own results and, if necessary, seek expert advice.

Publication of your CIF in IUCr journals

A basic structural check has been run on your CIF. These basic checks will be run on all CIFs submitted for publication in IUCr journals (*Acta Crystallographica*, *Journal of Applied Crystallography*, *Journal of Synchrotron Radiation*); however, if you intend to submit to *Acta Crystallographica Section C* or *E* or *IUCrData*, you should make sure that full publication checks are run on the final version of your CIF prior to submission.

Publication of your CIF in other journals

Please refer to the *Notes for Authors* of the relevant journal for any special instructions relating to CIF submission.

PLATON version of 13/07/2021; check.def file version of 13/07/2021

Datablock cu_191120_gs_isonaft_0m_a - ellipsoid plot

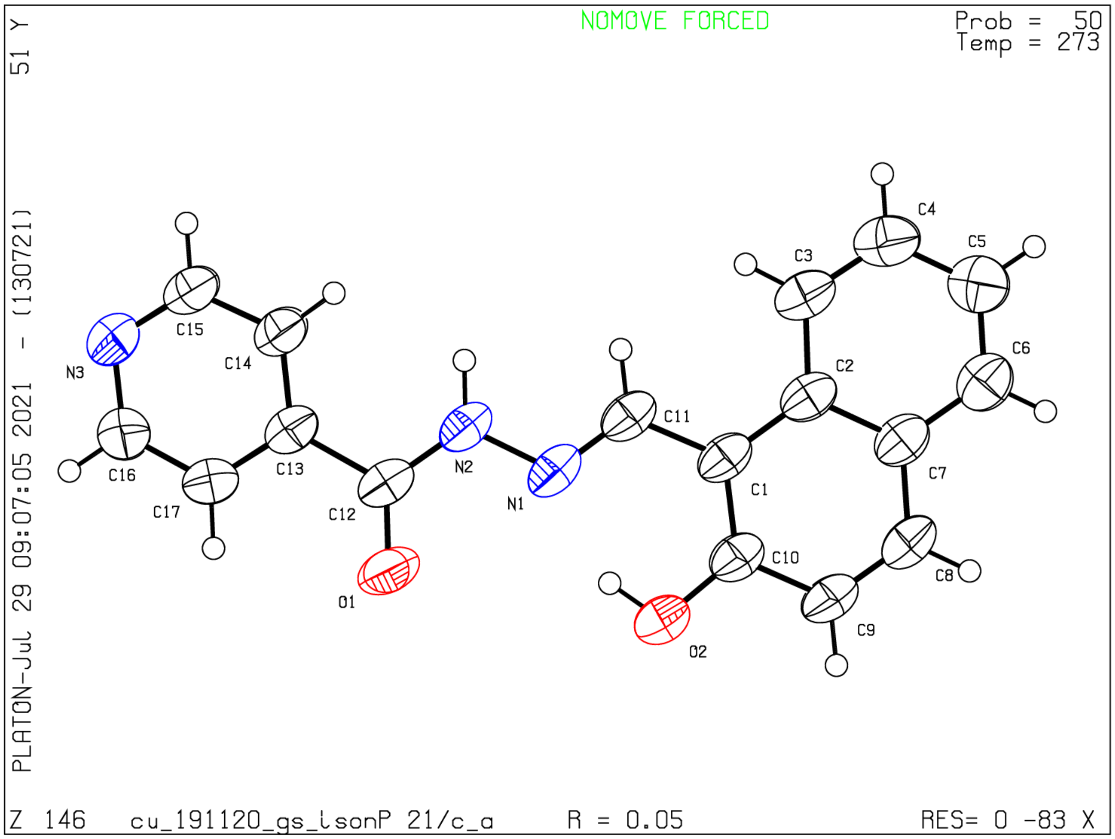

Supplement: Supplementary file 1 [file molecules-26-05375-s001.zip › molecules-1344319(Supplementary)/checkcif_IN.pdf]
