# Supplementary material for: Heteroleptic Oxidovanadium(V) Complexes with Activity against Infective and Non-Infective Stages of Trypanosoma cruzi
Source: Molecules. 2021 Sep 3;26(17):5375. doi: 10.3390/molecules26175375 (PMC8433833; doi:10.3390/molecules26175375)

checkCIF/PLATON report

Structure factors have been supplied for datablock(s) cu_191121_GS

THIS REPORT IS FOR GUIDANCE ONLY. IF USED AS PART OF A REVIEW PROCEDURE FOR PUBLICATION, IT SHOULD NOT REPLACE THE EXPERTISE OF AN EXPERIENCED CRYSTALLOGRAPHIC REFEREE.

No syntax errors found. CIF dictionary Interpreting this report

Datablock: cu_191121_GS

Bond precision: C-C = 0.0085 A Wavelength=1.54178

Cell:	a=22.3574(11)	b=16.3235(8)	c=16.9294(8)
	alpha=90	beta=90	gamma=90
Temperature:	273 K		

	Calculated	Reported
Volume	6178.4(5)	6178.4(5)
Space group	P c c n	P c c n
Hall group	-P 2ab 2ac	-P 2ab 2ac
Moiety formula	2(C26 H15 Cl I N4 O4 V), 3(C4 H8 O)	?
Sum formula	C64 H54 Cl2 I2 N8 O11 V2	C32 H27 Cl I N4 O5.50 V
Mr	1537.73	768.86
Dx,g cm-3	1.653	1.653
Z	4	8
Mu (mm-1)	11.744	11.744
F000	3072.0	3072.0
F000’	3081.29	
h,k,lmax	28,20,21	28,20,21
Nref	6774	6718
Tmin,Tmax	0.127,0.253	0.421,0.754
Tmin’	0.057	

Correction method= # Reported T Limits: Tmin=0.421 Tmax=0.754
AbsCorr = MULTI-SCAN

Data completeness= 0.992 Theta(max)= 80.490

R(reflections)= 0.0563(4416) wR2(reflections)= 0.1782(6718)

S = 1.056 Npar= 471

The following ALERTS were generated. Each ALERT has the format
test-name_ALERT_alert-type_alert-level.
Click on the hyperlinks for more details of the test.

Alert level C

```
PLAT260_ALERT_2_C Large Average Ueq of Residue Including       O5A      0.123 Check
PLAT260_ALERT_2_C Large Average Ueq of Residue Including       O5B      0.223 Check
PLAT260_ALERT_2_C Large Average Ueq of Residue Including       O5C      0.223 Check
PLAT342_ALERT_3_C Low Bond Precision on  C-C Bonds ...............    0.00846 Ang.
PLAT906_ALERT_3_C Large K Value in the Analysis of Variance ......      2.782 Check
```

Alert level G

```
PLAT002_ALERT_2_G Number of Distance or Angle Restraints on AtSite         15 Note
PLAT003_ALERT_2_G Number of Uiso or Uij Restrained non-H Atoms ...         15 Report
PLAT045_ALERT_1_G Calculated and Reported Z Differ by a Factor ...       0.50 Check
PLAT083_ALERT_2_G SHELXL Second Parameter in WGHT  Unusually Large       9.23 Why ?
PLAT172_ALERT_4_G The CIF-Embedded .res File Contains DFIX Records          2 Report
PLAT175_ALERT_4_G The CIF-Embedded .res File Contains SAME Records          4 Report
PLAT178_ALERT_4_G The CIF-Embedded .res File Contains SIMU Records          2 Report
PLAT199_ALERT_1_G Reported _cell_measurement_temperature ..... (K)        273 Check
PLAT200_ALERT_1_G Reported   _diffrn_ambient_temperature ..... (K)        273 Check
PLAT300_ALERT_4_G Atom Site Occupancy of O5A        Constrained at        0.5 Check
PLAT300_ALERT_4_G Atom Site Occupancy of C1A        Constrained at        0.5 Check
PLAT300_ALERT_4_G Atom Site Occupancy of C2A        Constrained at        0.5 Check
PLAT300_ALERT_4_G Atom Site Occupancy of C3A        Constrained at        0.5 Check
PLAT300_ALERT_4_G Atom Site Occupancy of C4A        Constrained at        0.5 Check
PLAT300_ALERT_4_G Atom Site Occupancy of H1AA       Constrained at        0.5 Check
PLAT300_ALERT_4_G Atom Site Occupancy of H1AB       Constrained at        0.5 Check
PLAT300_ALERT_4_G Atom Site Occupancy of H2AA       Constrained at        0.5 Check
PLAT300_ALERT_4_G Atom Site Occupancy of H2AB       Constrained at        0.5 Check
PLAT300_ALERT_4_G Atom Site Occupancy of H3AA       Constrained at        0.5 Check
PLAT300_ALERT_4_G Atom Site Occupancy of H3AB       Constrained at        0.5 Check
PLAT300_ALERT_4_G Atom Site Occupancy of H4AA       Constrained at        0.5 Check
PLAT300_ALERT_4_G Atom Site Occupancy of H4AB       Constrained at        0.5 Check
PLAT302_ALERT_4_G Anion/Solvent/Minor-Residue Disorder (Resd  2  )       100% Note
PLAT302_ALERT_4_G Anion/Solvent/Minor-Residue Disorder (Resd  3  )       100% Note
PLAT302_ALERT_4_G Anion/Solvent/Minor-Residue Disorder (Resd  4  )       100% Note
PLAT304_ALERT_4_G Non-Integer Number of Atoms in ..... (Resd  2  )       6.50 Check
PLAT304_ALERT_4_G Non-Integer Number of Atoms in ..... (Resd  3  )       6.73 Check
PLAT304_ALERT_4_G Non-Integer Number of Atoms in ..... (Resd  4  )       6.27 Check
PLAT398_ALERT_2_G Deviating  C-O-C    Angle From 120 for O5A             99.2 Degree
PLAT398_ALERT_2_G Deviating  C-O-C    Angle From 120 for O5B             99.1 Degree
PLAT398_ALERT_2_G Deviating  C-O-C    Angle From 120 for O5C             98.1 Degree
PLAT411_ALERT_2_G Short Inter H...H Contact  H3       ..H3CB     .       1.73 Ang.
                                             x,3/2-y,-1/2+z  =      7_575 Check
PLAT411_ALERT_2_G Short Inter H...H Contact  H4AA     ..H18      .       1.96 Ang.
                                                      x,y,z  =      1_555 Check
PLAT411_ALERT_2_G Short Inter H...H Contact  H4AB     ..H18      .       1.63 Ang.
                                              1/2-x,3/2-y,z  =      2_565 Check
PLAT431_ALERT_2_G Short Inter HL..A Contact  I1       ..N2       .       3.21 Ang.
                                             x,3/2-y,-1/2+z  =      7_575 Check
PLAT720_ALERT_4_G Number of Unusual/Non-Standard Labels ..........         29 Note
PLAT721_ALERT_1_G Bond    Calc     0.96000, Rep     0.97000 Dev...       0.01 Ang.
              C2A      -H2AA           1.555   1.555 ........     #   66 Check
PLAT789_ALERT_4_G Atoms with Negative _atom_site_disorder_group  #         13 Check
PLAT860_ALERT_3_G Number of Least-Squares Restraints .............        252 Note
PLAT883_ALERT_1_G No Info/Value for _atom_sites_solution_primary .     Please Do !
PLAT912_ALERT_4_G Missing # of FCF Reflections Above STh/L=  0.600         57 Note
PLAT978_ALERT_2_G Number C-C Bonds with Positive Residual Density.          2 Info
```

```
PLAT992_ALERT_5_G Repd & Actual _reflns_number_gt Values Differ by          1 Check
```

```
   0 ALERT level A = Most likely a serious problem - resolve or explain
   0 ALERT level B = A potentially serious problem, consider carefully
   5 ALERT level C = Check. Ensure it is not caused by an omission or oversight
  43 ALERT level G = General information/check it is not something unexpected

   5 ALERT type 1 CIF construction/syntax error, inconsistent or missing data
  14 ALERT type 2 Indicator that the structure model may be wrong or deficient
   3 ALERT type 3 Indicator that the structure quality may be low
  25 ALERT type 4 Improvement, methodology, query or suggestion
   1 ALERT type 5 Informative message, check
```

Validation response form

Please find below a validation response form (VRF) that can be filled in and pasted into your CIF.

```
# start Validation Reply Form
_vrf_PLAT260_cu_191121_GS
;
PROBLEM: Large Average Ueq of Residue Including       O5A      0.123 Check
RESPONSE: ...
;
_vrf_PLAT342_cu_191121_GS
;
PROBLEM: Low Bond Precision on  C-C Bonds ...............    0.00846 Ang.
RESPONSE: ...
;
_vrf_PLAT906_cu_191121_GS
;
PROBLEM: Large K Value in the Analysis of Variance ......      2.782 Check
RESPONSE: ...
;
# end Validation Reply Form
```

It is advisable to attempt to resolve as many as possible of the alerts in all categories. Often the minor alerts point to easily fixed oversights, errors and omissions in your CIF or refinement strategy, so attention to these fine details can be worthwhile. In order to resolve some of the more serious problems it may be necessary to carry out additional measurements or structure refinements. However, the purpose of your study may justify the reported deviations and the more serious of these should normally be commented upon in the discussion or experimental section of a paper or in the "special_details" fields of the CIF. checkCIF was carefully designed to identify outliers and unusual parameters, but every test has its limitations and alerts that are not important in a particular case may appear. Conversely, the absence of alerts does not guarantee there are no aspects of the results needing attention. It is up to the individual to critically assess their own results and, if necessary, seek expert advice.

Publication of your CIF in IUCr journals

A basic structural check has been run on your CIF. These basic checks will be run on all CIFs submitted for publication in IUCr journals (*Acta Crystallographica*, *Journal of Applied Crystallography*, *Journal of Synchrotron Radiation*); however, if you intend to submit to *Acta Crystallographica Section C* or *E* or *IUCrData*, you should make sure that full publication checks are run on the final version of your CIF prior to submission.

Publication of your CIF in other journals

Please refer to the *Notes for Authors* of the relevant journal for any special instructions relating to CIF submission.

PLATON version of 22/12/2019; check.def file version of 13/12/2019

Datablock cu_191121_GS - ellipsoid plot

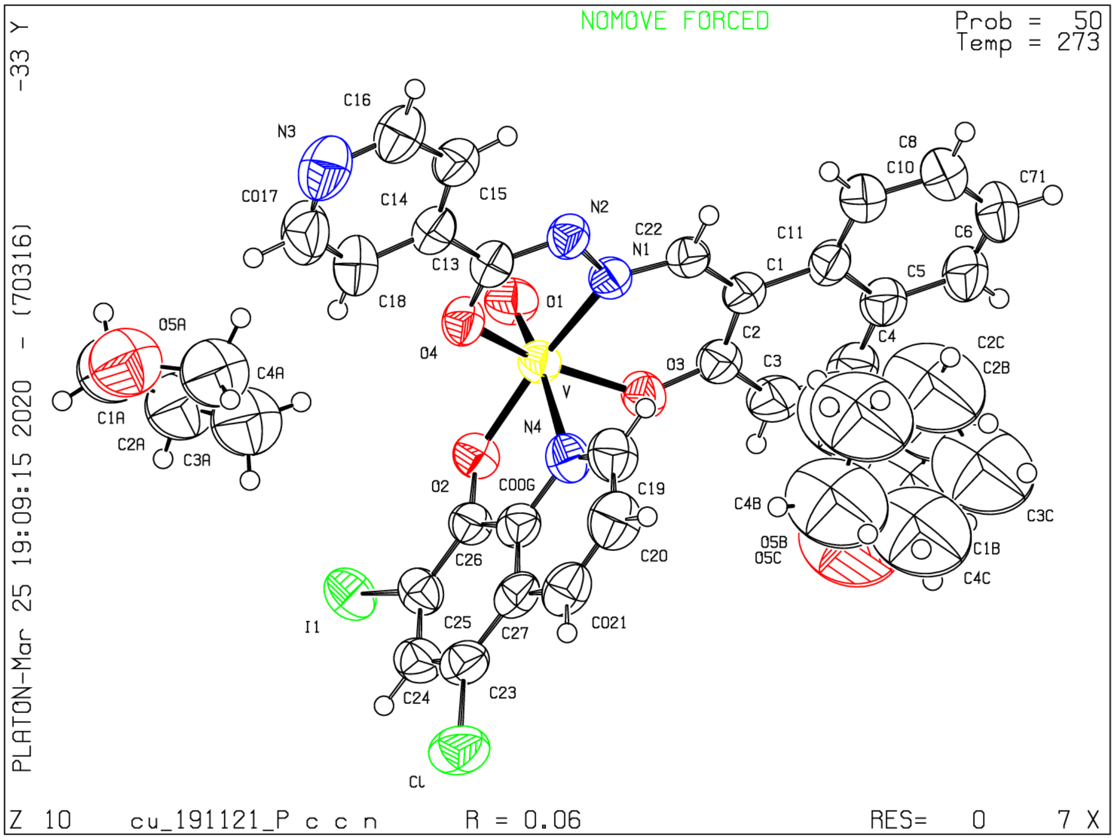

Supplement: Supplementary file 1 [file molecules-26-05375-s001.zip › molecules-1344319(Supplementary)/checkcif_VOHQClIIN.pdf]
