# Supplementary material for: Heteroleptic Oxidovanadium(V) Complexes with Activity against Infective and Non-Infective Stages of Trypanosoma cruzi
Source: Molecules. 2021 Sep 3;26(17):5375. doi: 10.3390/molecules26175375 (PMC8433833; doi:10.3390/molecules26175375)
Supplement: Supplementary file 1 [file molecules-26-05375-s001.zip › molecules-1344319(Supplementary)/molecules-1344319(supplementary).pdf]

molecules

Supplementary Material

Heteroleptic Oxidovanadium(V) Complexes with Activity Against Infective and Non-Infective Stages of *Trypanosoma cruzi*

Gonzalo Scalese [1,2], Ignacio Machado [3], Gustavo Salinas [4,5], Leticia Pérez-Díaz [6] and Dinorah Gambino [1,*]

[1] Área Química Inorgánica, Facultad de Química, Universidad de la República, 11800 Montevideo, Uruguay; gscalese@fq.edu.uy
[2] Programa de Posgrados de la Facultad de Química, Universidad de la República, 11800 Montevideo, Uruguay
[3] Área Química Analítica, Facultad de Química, Universidad de la República, 11800 Montevideo, Uruguay; imachado@fq.edu.uy
[4] Worm Biology Lab, Institut Pasteur de Montevideo, 1400 Montevideo, Uruguay; gsalinas@pasteur.edu.uy
[5] Departamento de Biociencias, Facultad de Química, 11800 Montevideo, Uruguay; gsalinas@pasteur.edu.uy
[6] Laboratorio de Interacciones Moleculares, Facultad de Ciencias, Universidad de la República, 1400 Montevideo, Uruguay; lperez@fcien.edu.uy
* Correspondence: dgambino@fq.edu.uy; Tel.: +598 29249739

Table S1. Tentative assignment of selected IR bands of the [V^VO(IN–2H)(L-H)] complexes. Bands for the free ligands L0-L4, IN and the [V^VO_2(IN–H)] are included for comparison. Band positions are given in cm^{-1}.

Compound	ν(VO)	ν(Ar-OH)	ν(NH)	ν(CO)	ν(CN)
IN	-	3213 m	3038 m	1680 s	1575 s
[V^VO_2(IN–H)]	915 s; 827 m	-	3074 w	1633 m	1597 s
L0[a]	-	3159 b	-	-	1624 vw
[V^VO(IN–2H)(L0-H)]	974 s	-	-	1620 w	1594 s
L1[a]	-	3132 b	-	-	1615 w
[V^VO(IN–2H)(L1-H)]	978 s	-	-	1618 w	1593s
L2[a]	-	3132 b	-	-	1605 w
[V^VO(IN–2H)(L2-H)]	972 s	-	-	1618 w	1593 s
L3[a]	-	3026 b	-	-	1576 vw
[V^VO(IN–2H)(L3-H)]	966 s	-	-	1620 w	1593 s
L4[b]	-	3235 b	-	-	1628 vw
[V^VO(IN–2H)(L4-H)]	968 s	-	-	1618 w	1593 m

s:strong; w: weak; vw: very weak; m: medium; b: broad; sh; shoulder

[a] data from reference [44]

[b] data from reference [42]

Table S2. Selected bond lengths [Å] and angles around vanadium(V) [°] in [V^{V}O(IN-2H)(L2-H)]·1.5 THF

Bond distances	
V-O(1)	1.590(4)
V-O(2)	1.853(4)
V-O(3)	1.845(4)
V-O(4)	1.931(4)
V-N(1)	2.077(4)
V-N(4)	2.390(4)
**Bond angles**	
O(1)-V-O(2)	100.28(18)
O(1)-V-O(3)	100.40(2)
O(2)-V-O(3)	100.89(17)
O(1)-V-O(4)	97.78(19)
O(2)-V-O(4)	94.58(16)
O(3)-V-O(4)	153.51(18)
O(1)-V-N(1)	99.07(19)
O(2)-V-N(1)	159.31(16)
O(3)-V-N(1)	82.65(17)
O(4)-V-N(1)	75.57(16)
O(1)-V-N(4)	174.39(19)
O(2)-V-N(4)	75.21(15)
O(3)-V-N(4)	83.86(18)
O(4)-V-N(4)	79.45(16)
N(1)-V-N(4)	85.01(16)

Table S3. ^{1}H-NMR signals of the [V^VO(IN–2H)(L–H)] complexes in DMSO-d_6 at 25°C (δ, ppm). Free ligands are included for comparison.

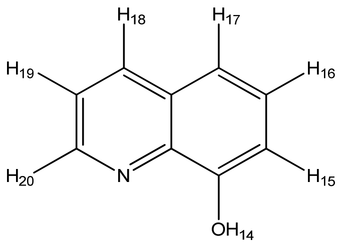

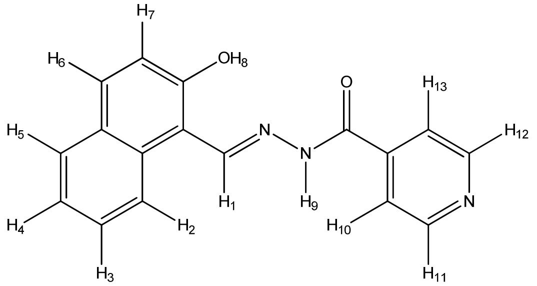

H	[V^VO(IN–2H)(L0–H)]			[V^VO(IN–2H)(L1–H)]			[V^VO(IN–2H)(L2–H)]			[V^VO(IN–2H)(L3–H)]]			[V^VO(IN–2H)(L4–H)]]		
	δ_{ligand}	$\delta_{complex}$	Δδ	δ_{ligand}	$\delta_{complex}$	Δδ	δ_{ligand}	$\delta_{complex}$	Δδ	δ_{ligand}	$\delta_{complex}$	Δδ	δ_{ligand}	$\delta_{complex}$	Δδ
**1**	9.48	10.12	0.64	9.48	10.15	0.67	9.48	10.14	0.66	9.48	10.14	0.66	9.48	10.18	0.70
**2**	8.32	8.68	0.36	8.32	8.66	0.34	8.32	8.63	0.31	8.32	8.62	0.30	8.32	8.67	0.35
**3**	7.62	7.77	0.15	7.62	7.75	0.13	7.62	7.75	0.13	7.62	7.75	0.13	7.62	7.76	0.14
**4**	7.42	7.58	0.16	7.42	7.54	0.12	7.42	7.54	0.12	7.42	7.54	0.12	7.42	7.56	0.14
**5**	7.89	7.98	0.09	7.89	7.98	0.09	7.89	7.98	0.09	7.89	7.98	0.09	7.89	8.00	0.11
**6**	7.95	8.17	0.22	7.95	8.21	0.26	7.95	8.20	0.25	7.95	8.20	0.25	7.95	8.23	0.28
**7**	7.25	7.10	−0.15	7.25	7.14	−0.11	7.25	7.14	−0.11	7.25	7.12	−0.13	7.25	7.14	−0.11
**8**	12.53	-	-	12.53	-	-	12.53	-	-	12.53	-	-	12.53	-	-
**9**	12.40	-	-	12.40	-	-	12.40	-	-	12.40	-	-	12.40	-	-
**10**	7.89	7.62	−0.19	7.89	7.62	−0.27	7.89	7.61	−0.28	7.89	7.61	−0.28	7.89	7.61	−0.28
**11**	8.84	8.62	−0.22	8.84	8.61	−0.23	8.84	8.63	−0.21	8.84	8.62	−0.22	8.84	8.62	−0.22
**12**	8.84	8.62	−0.22	8.84	8.61	−0.23	8.84	8.63	−0.21	8.84	8.62	−0.22	8.84	8.62	−0.22
**13**	7.89	7.62	−0.19	7.89	7.62	−0.27	7.89	7.61	−0.28	7.89	7.61	−0.28	7.89	7.61	−0.28
**14**	9.82	-	-	11.00	-	-	11.07	-	-	*	-	-	*	-	-
**15**	7.09	7.77	0.68	-	-	-	-	-	-	-	-	-	7.20	7.46	0.26
**16**	7.41	7.20	−0.21	7.82	8.19	0.37	8.00	8.35	0.35	8.34	8.69	0.35	8.55	8.78	0.23
**17**	7.41	7.77	0.36	-	-	-	-	-	-	-	-	-	-	-	-
**18**	8.32	8.55	0.22	8.50	8.61	0.11	8.50	8.57	0.07	8.29	8.35	0.06	9.15	9.15	0.00
**19**	7.53	7.60	−0.07	7.85	7.67	−0.15	7.77	7.67	−0.10	7.73	7.63	−0.10	7.89	7.80	−0.09
**20**	8.85	8.22	−0.63	9.00	8.34	−0.65	8.97	8.29	−0.68	8.87	8.20	−0.67	9.02	8.36	−0.66

$\Delta\delta = \delta_{complex} - \delta_{ligand}$ *Not observed

Table S4. Crystal data and structure refinement results for IN and [V^{V}O(IN–2H)(L2–H)]·1.5 THF

	**IN**	**[V^{V}O(IN–2H)(L2–H)]·1.5 THF**
Formula	$C_{17}H_{13}N_3O_2$	$C_{32}H_{27}ClIN_4O_{5.5}V$
Formula weight	291.30	768.86
Temperature (K)	273(2)	273(2)
Wavelength (Å)	CuKα (λ = 1.54178)	CuKα (λ = 1.54178)
Crystal system	monoclínic	orthorrombic
Space group	$P2_1/c$	Pccn
Unit cell dimensions		
a (Å)	8.786(2)	22.3574(11)
b (Å)	10.252(2)	16.3235(8)
c (Å)	15.793(5)	16.9294(8)
β (°)	99.423(10)	
Volume (Å^3)	1403.5(7)	6178.4(5)
Z, density (calculated, g cm^{-3})	4; 1.379	8; 1.653
Absorption coefficient (mm^{-1})	0.759	11.744
F(000)	608.0	3072.0
Crystal shape/color	Needle / yellow	Needle / dark purple
Crystal size (mm^3)	0.326 × 0.233 × 0.155	0.210 × 0.180 × 0.117
ϑ-range (º) for data collection	10.2 to 160.4	6.704 to 160.98
Index ranges	−11 ≤ h ≤ 11, −12 ≤ k ≤ 12, −19 ≤ l ≤ 19	−28 ≤ h ≤ 21, −20 ≤ k ≤ 20, −21 ≤ l ≤ 19
Reflections collected	22284	60109
Independent reflections	3009 [R_{int} = 0.0376, R_{sigma} = 0.0273]	6718 [R_{int} = 0.0823, R_{sigma} = 0.0409]
Observed reflections [I>2σ(I)]	3009	6718
Completeness (%)	98.4	99.2
Absorption correction	multi-scan	multi-scan
Max. and min. transmission	0.7543 and 0.6386	0.7538 and 0.4239
Refinement method	Full-matrix least-squares on F^2	Full-matrix least-squares on F^2
Data / restraints / parameters	2009/0/208	6718/252/471
Goodness-of-fit on F^2	1.033	1.056
Final R indices[a] [I>2σ(I)]	R_1 = 0.0470, wR_2 = 0.1303	R_1 = 0.0563, wR_2 = 0.1505
R indices (all data)	R_1 = 0.0556, wR_2 = 0.1404	R_1 = 0.0895, wR_2 = 0.1782
Largest diff. peak and hole (e.Å^{-3})	0.21/-0.23	0.85/-1.05

[a] $R_1=\Sigma||F_o|-|F_c||/\Sigma|F_o|$, $wR_2=[\Sigma w(|F_o|^2-|F_c|^2)^2/\Sigma w(|F_o|^2)^2]^{1/2}$

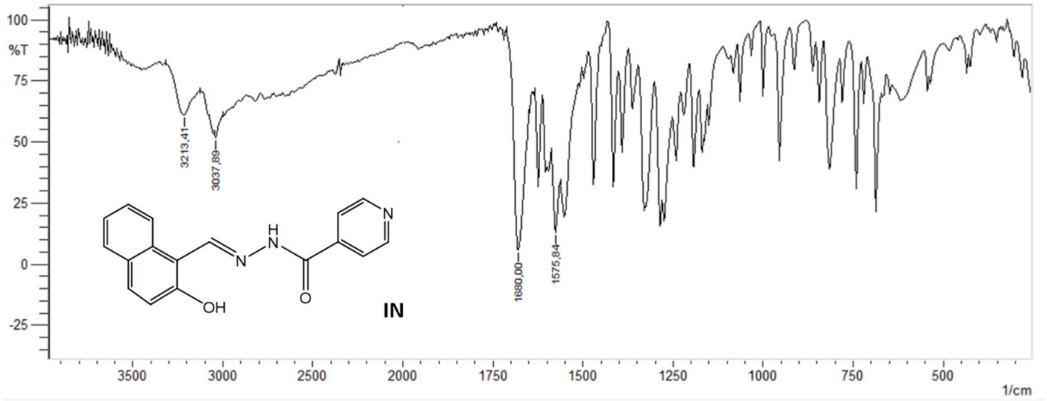

Figure S1. Infrared spectra of IN free ligand (4000–400 cm^{-1}).

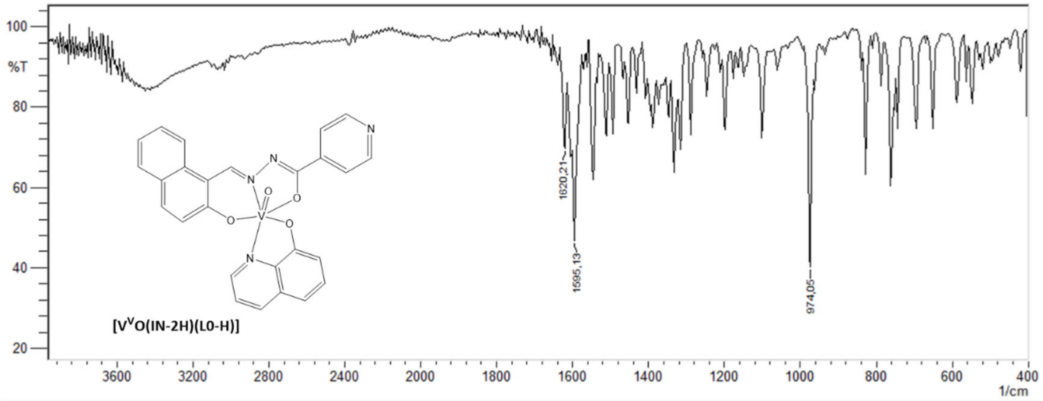

Figure S2. Infrared spectra of [VO(IN-2H)(L0-H)] (4000–400 cm^{-1}).

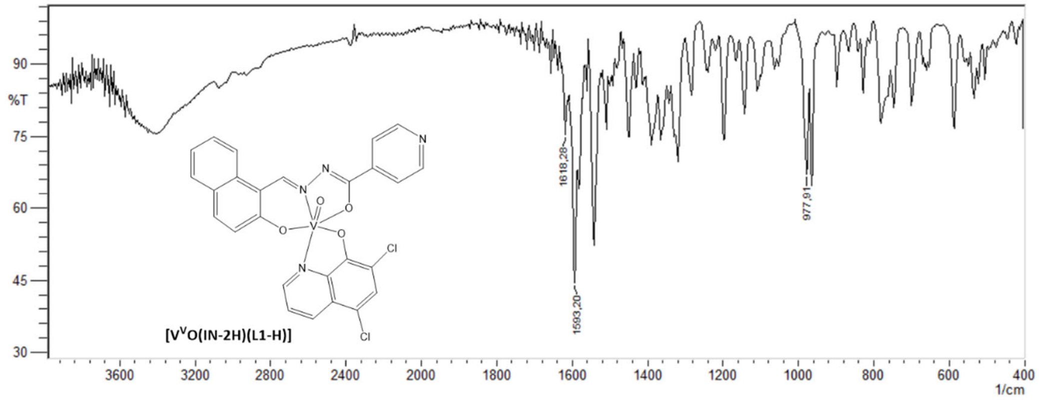

Figure S3. Infrared spectra of [VO(IN-2H)(L1-H)] (4000–400 cm^{-1}).

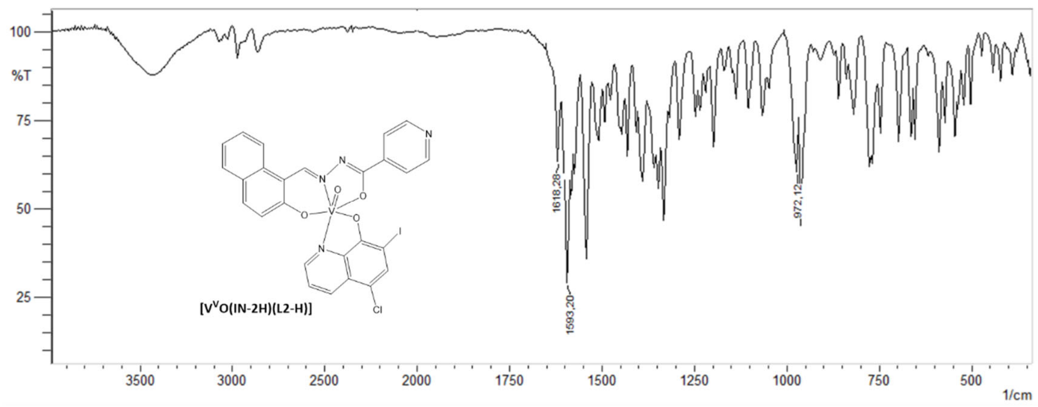

Figure S4. Infrared spectra of [VO(IN-2H)(L2-H)] (4000–400 cm^{-1}).

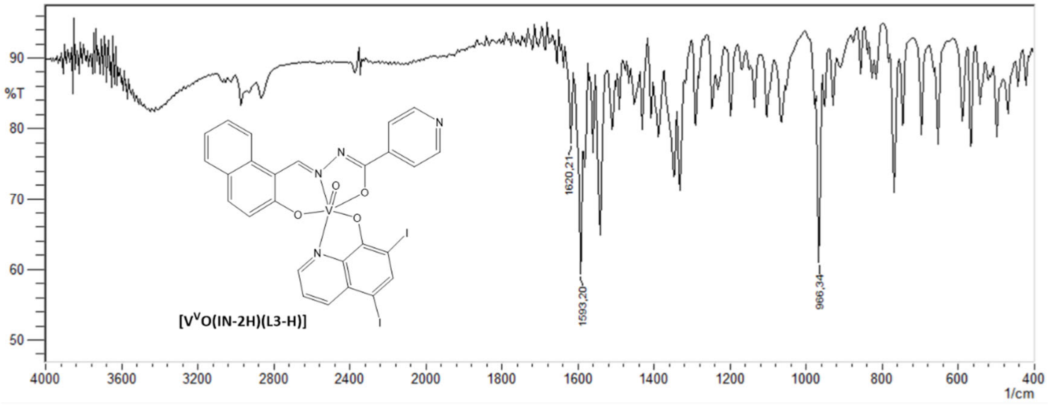

Figure S5. Infrared spectra of [VO(IN-2H)(L3-H)] (4000–400 cm^{-1}).

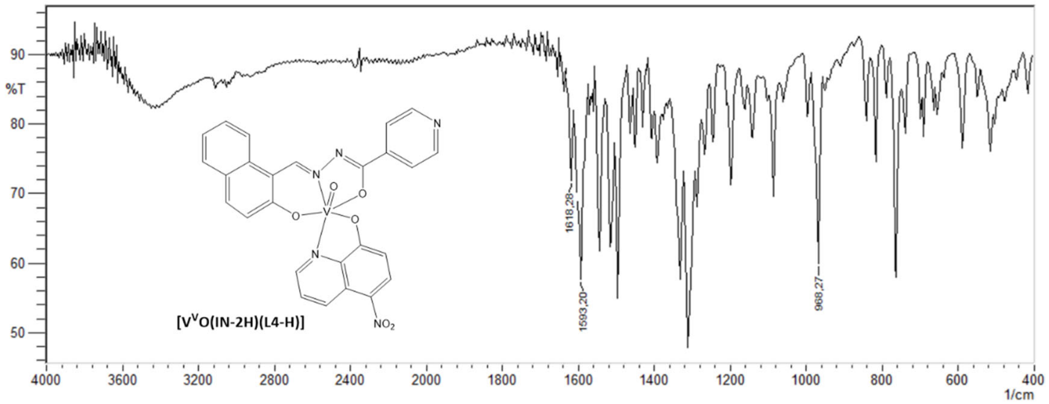

Figure S6. Infrared spectra of [VO(IN-2H)(L4-H)] (4000–400 cm^{-1}).

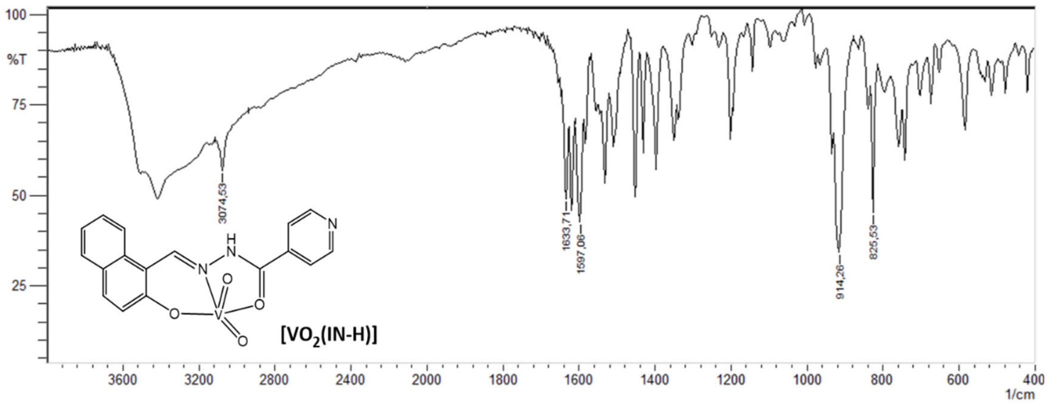

Figure S7. Infrared spectra of [VO(IN-2H)(L4-H)] (4000–400 cm[−1]).

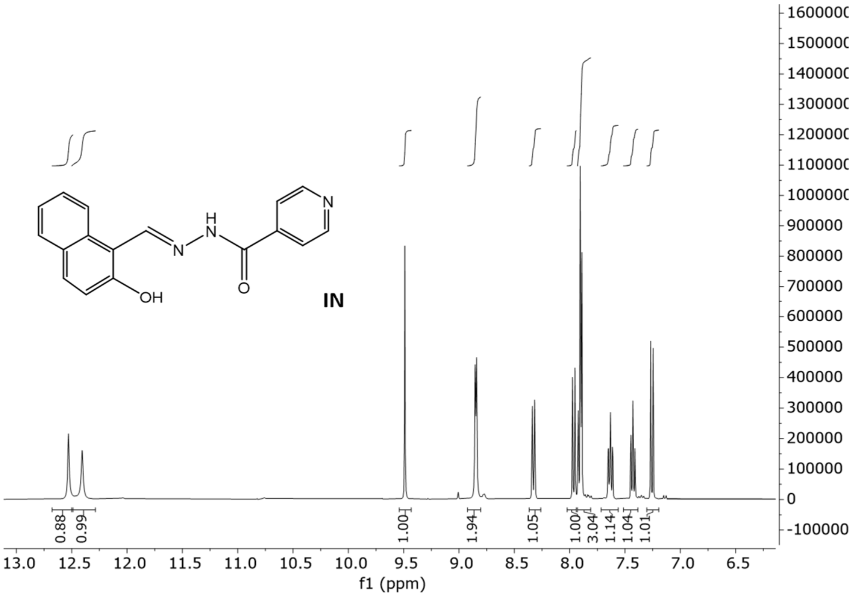

Figure S8. ^{1}H-NMR spectra for IN free ligand

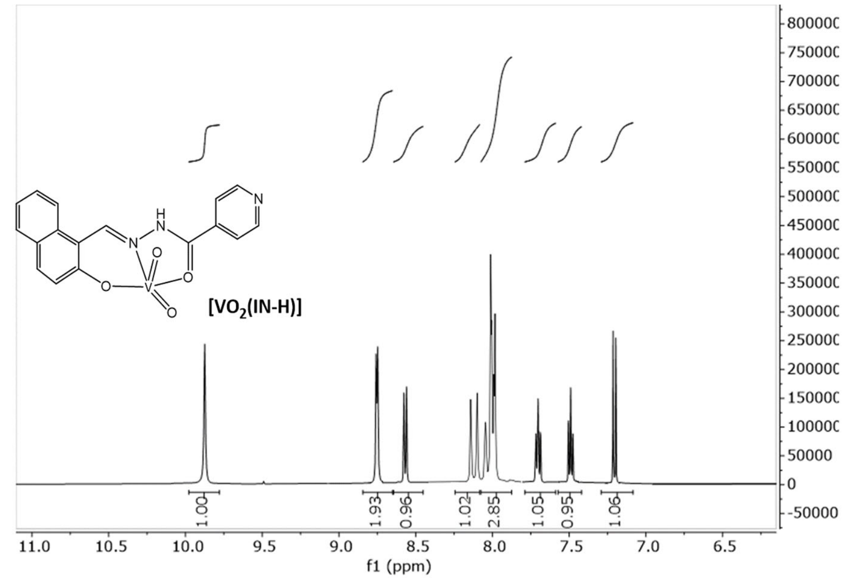

Figure S9. ^{1}H-NMR spectra) for [VO_2(IN-H)]

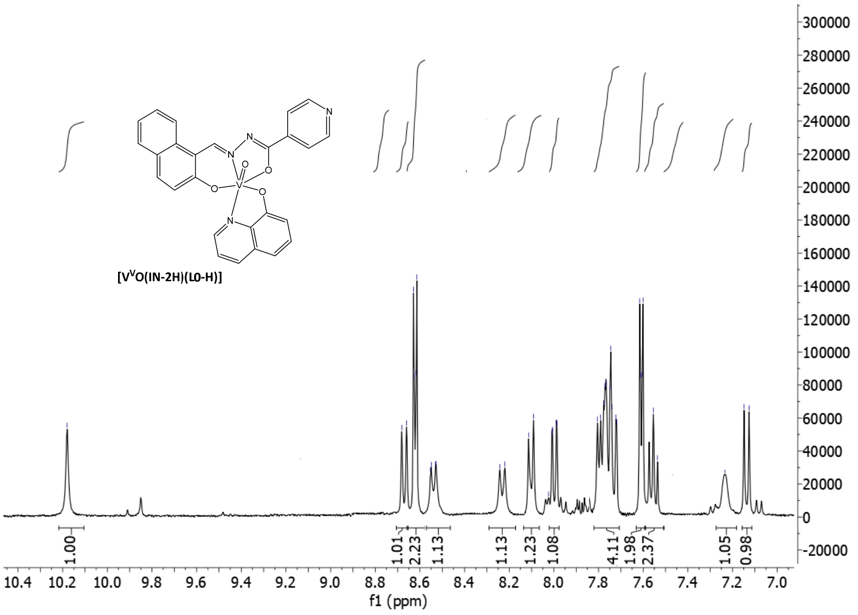

Figure S10. ^{1}H-NMR spectra for [VO(IN-2H)(L0-H)]

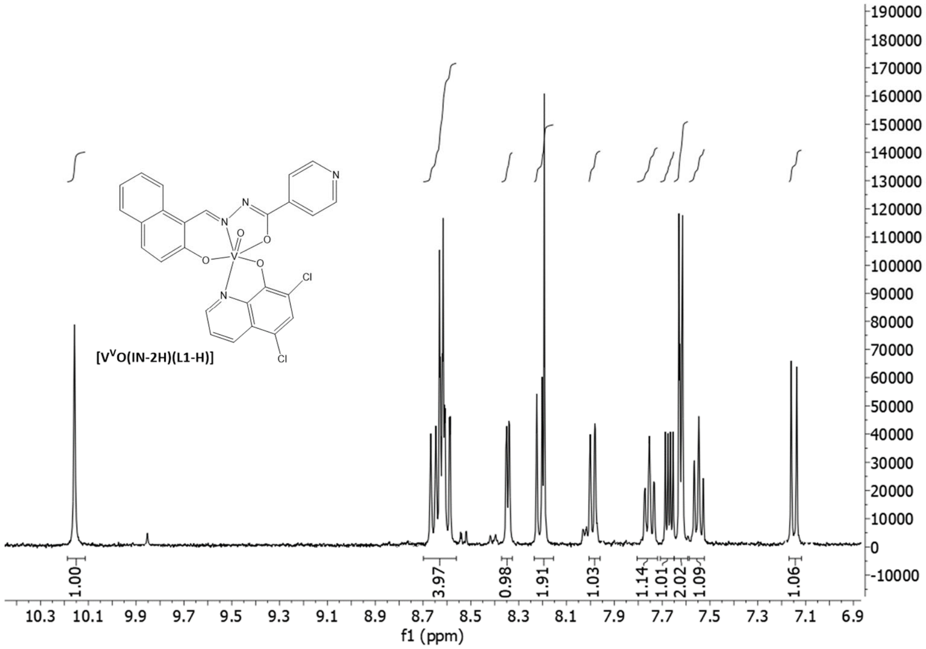

Figure S11. ^{1}H-NMR spectra for [VO(IN-2H)(L1-H)]

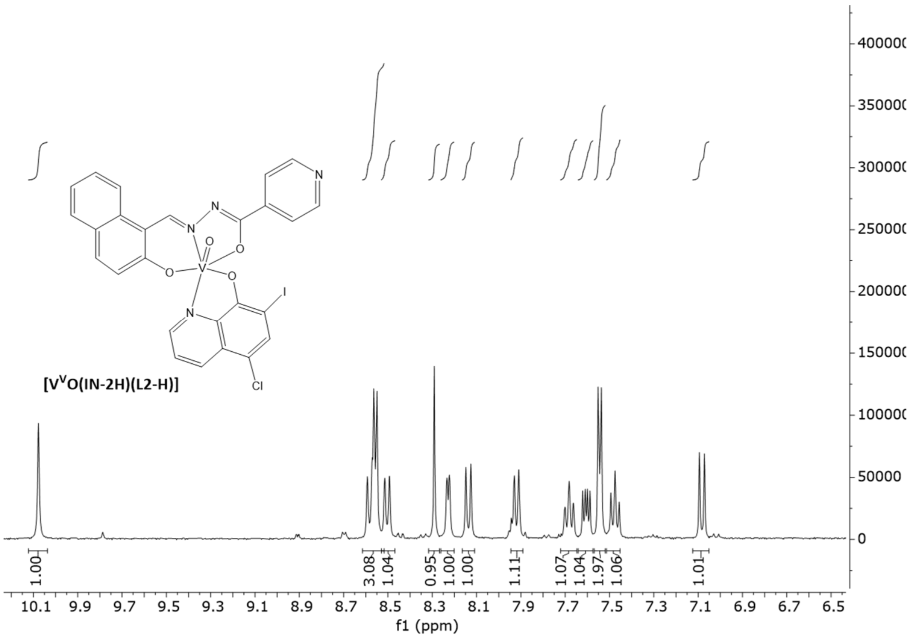

Figure S12. ^{1}H-NMR spectra for [VO(IN-2H)(L2-H)

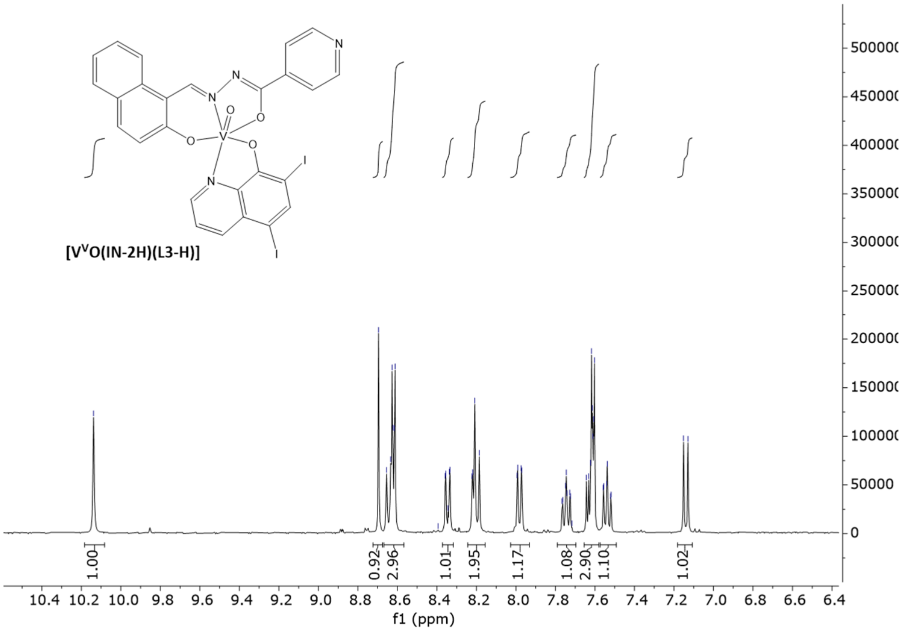

Figure S13. ^{1}H-NMR spectra for [VO(IN-2H)(L3-H)]

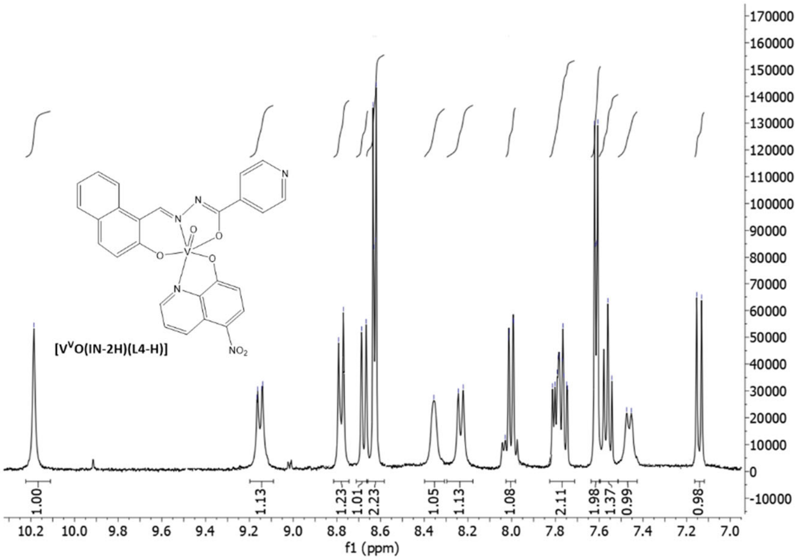

Figure S14. ^{1}H-NMR spectra for [VO(IN-2H)(L4-H)]

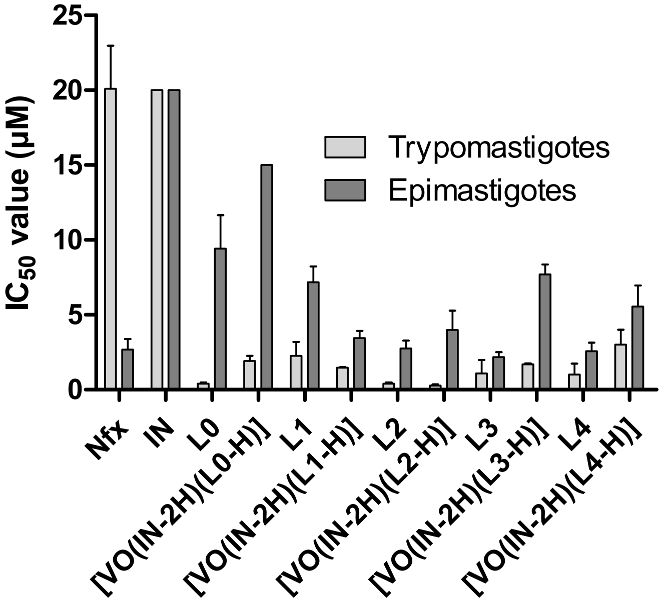

Figure S15. IC_{50} values on epimastigotes and trypomastigotes of *T. cruzi* of the new V^VO-complexes comparing to free ligands and reference drug Nifurtimox (Nfx).
